# Sex-specific gonadal transcriptome during early development of Siberian sturgeon

**DOI:** 10.1186/s13293-025-00810-8

**Published:** 2026-02-02

**Authors:** André Lasalle Gerla, Germán Benech-Correa, Christophe Klopp, Denise Vizziano-Cantonnet

**Affiliations:** 1 Laboratorio de Fisiología de la Reproducción y Ecología de Peces, Instituto de Biología, Facultad de Ciencias, Universidad de la República Oriental del Uruguay, Iguá, Montevideo, 4225, 11400 Uruguay; 2 INRA, SIGENAE, Genotoul Bioinfo, MIAT UR875, CASTANET-TOLOSAN CEDEX, Chemin de Borde-Rouge - Auzeville BP 52627, 31326 Auzeville-Tolosane, France

**Keywords:** Siberian sturgeon, Sex differentiation, Gonadal development, Gonadal transcriptome, Differential gene expression

## Abstract

**Background:**

Sex determination and differentiation are complex processes shaped by a wide variety of molecular factors. In contrast to teleost species, many aspects of these processes remain poorly understood in basal non-teleost fishes such as the Siberian sturgeon (*Acipenser baerii*). Genetic sexing of this important aquaculture species now enables studies of undifferentiated males and females to identify factors involved in early sexual differentiation.

**Methods:**

Twelve undifferentiated Siberian sturgeon (six males, six females) were genetically sexed at 2.5 months of age. High-quality RNA was extracted from gonad samples, and transcriptomes were assembled using a reference dataset. Bioinformatic analyses were performed to identify sex-biased genes through differential expression analysis, Gene Ontology (GO) enriched terms, and classification of coding and non-coding sequences.

**Results:**

Genes potentially associated with sex differentiation were identified in gonadal tissue. Female-biased genes included classical estrogens production genes (*hsd17b1*, *cyp19a1*, *foxl2*) and immediate early response genes known to react rapidly to estrogens (*jun-b*, *c-fos*, *egr1*), as well as genes not previously linked to estradiol (*di-ras2*, *ier2*, *aanat*). The enriched Gene Ontology results suggested that melatonin signaling and hypothalamic pathways may also contribute to female differentiation. In males, the well-known factor *tbx1* was upregulated along with novel candidates (*plin1*,* nrxn3*,* chs2*,* mmp9*). No sex-biased genes related to androgen production were identified.

**Conclusion:**

By 2.5 months of age, sex-specific gonadal differences are already apparent in Siberian sturgeon. This study highlights the estrogen response pathway, including immediate early response genes described here for the first time in the context of fish gonadal differentiation. At the same time, an estrogen-independent ovarian pathway cannot be ruled out. Male differentiation appears to involve *tbx1* together with new candidates for testis regulation in the absence of sex-biased androgen-producing enzymes. These novel genes expressed near the onset of sex differentiation merit further investigation.

**Supplementary Information:**

The online version contains supplementary material available at 10.1186/s13293-025-00810-8.

## Highlights

The first transcriptomic analysis of undifferentiated gonads in genetically sexed Siberian sturgeon reveals both established and newly identified genes activated near the onset of sex differentiation.

Ovarian differentiation involves both estrogen-dependent and estrogen-independent factors.

A novel set of male-biased genes expands the list of potential contributors to testis differentiation in fish.

**Supplementary Information:**

The online version contains supplementary material available at 10.1186/s13293-025-00810-8.

## Abstract (plain language summary)

In animals, the development of ovaries or testes is a key step in their growth. This process involves many complex biological events. For many modern fish species, scientists already know a great deal about how this happens. However, in ancient fish like the Siberian sturgeon, much less is understood. Our goal was to explore how this process begins during early development in this species.

We studied young sturgeons at two and a half months of age, before visible differences in their gonads appeared. Their sex was identified using genetic markers. We examined six males and six females, analyzing the activity of thousands of genes in their gonads using advanced sequencing technologies to see which genes were active early in sexual development.

The female fish showed activation of some genes that respond rapidly to the hormone estrogen, alongside the well-known genes that help produce estrogens. We also detected activation of other genes that do not appear to depend directly on estrogens. These results suggest that additional signals, such as the hormone melatonin and possibly brain-derived factors — may also influence female gonadal development.

In males, we identified new candidate genes that may play important roles in testis development. Interestingly, only one previously known male-related gene was activated, and we found no activation of genes involved in production of male hormones (androgens).

By 2.5 months of age, sturgeons are already beginning to show sex-specific differences in their gonads. Our study uncovered new candidate genes that could explain how sex differentiation occurs in sturgeon, providing a foundation for future research on this ancient fish.

**Supplementary Information:**

The online version contains supplementary material available at 10.1186/s13293-025-00810-8.

## Background

Sex determination and differentiation are highly complex biological processes that have been extensively studied in teleost species, but their mechanisms remain poorly understood in basal fishes such as the Siberian sturgeon (*Acipenser baerii*). Studying these processes in slow-developing fish like sturgeons offers a valuable opportunity to investigate conserved and divergent mechanisms of sex regulation in vertebrates.

In teleosts, sex determination can involve genetic, environmental, or mixed mechanisms. Several master sex-determining genes have been described [1, 2], but most are not conserved across species. In contrast, key genes involved in estrogens synthesis (*cyp19a1*, *foxl2*, *hsd17b1*) are broadly conserved among teleosts [3–7], supporting the view that estrogens are major inducers of sex differentiation in fish [8]. In the non-teleost Siberian sturgeon, these three genes are also activated very early in female gonads [9], months before the first signs of sexual differentiation appear [10].

Other genes such as Wnt family member 4 (*Wnt4*), Beta catenin (*Ctnnb1*), and R-spondin 1 (*Rspo1*), crucial in the female pathway in mammals [11], are also involved in sex differentiation in some fish [7]. However, these genes do not show sex-dimorphic expression in Siberian sturgeon during early molecular differentiation [9]. Similarly, in the male pathway, genes such as anti-Müllerian hormone (*amh*), doublesex and mab-3 related transcription factor 1 (*dmrt1*), SRY-box transcription factor 9 (*sox9*), and desert hedgehog (*dhh*) are well known to promote testis differentiation in many teleosts [6, 12] do not show sex-specific expression at this stage in Siberian sturgeon [9]. In contrast, gonadal soma-derived factor (*gsdf*), a gene expressed in teleost gonads [13–16] and involved in male differentiation [17–19], has been detected in Siberian sturgeon databases [20], and one copy is activated in the male program [21].

Like many other sturgeons, Siberian sturgeon exhibit relatively slow gonadal development and very late puberty [22]. Sexual differentiation occurs in the juvenile stage, at around 5–6 months of age [10, 23], several months after gonad formation at ~ 1 month. This extended window provides an opportunity to study molecular differentiation prior to morphological changes.

Because sexual differentiation involves multiple cell types and extensive cell–cell communication, we hypothesized that additional, as yet undescribed, genes contribute to early differentiation in sturgeon. To test this hypothesis, we performed transcriptomic analyses of undifferentiated gonads from 12 genetically sexed individuals (six males, six females) at 2.5 months of age, that is, 1.5 months after gonad formation and coinciding with the onset of molecular differentiation [9]. This approach allowed us to identify sex-biased expression patterns and candidate genes and molecular pathways that could contribute to the control of early sexual differentiation.

## Methods

### Fish maintenance and sampling

All experiments and animal maintenance were carried out in accordance with the Comisión Honoraria de Experimentación Animal (CHEA) protocol N° 1516 (exp. 240012-000023-22). Siberian sturgeon was provided by the Estuario del Plata sturgeon farm (Tacuarembó, Uruguay) and transported to the Facultad de Ciencias (Universidad de la República Oriental del Uruguay, Montevideo) aquaculture facilities at two months of age. Fish were maintained under natural photoperiod conditions, in a 500 L tank with 250 L of daily water renewal, at natural spring temperatures (17–21 °C). Fish were fed daily at 2% of body weight. Fin clips were collected at two months of age and preserved in 95% ethanol (PPA) until DNA extraction for sexing by PCR. Fish were then acclimated for 15 days before gonad sampling, performed on 12 genetically sexed males and females. Length (± 1 mm) and weight (± 0.01 g) were recorded (Additional File 1).

### Genomic DNA and RNA extraction

Genomic DNA extraction was performed following the laboratory protocol. Each Siberian sturgeon caudal fin sample (2 mm) was placed in a sterile 1.5 mL microcentrifuge tube. Twenty-five microliters of tissue digestion buffer (25 mM NaOH and 0.2 mM EDTA) were added to each sample and vortexed briefly. Samples were then heated for 15 min at 95 °C in a BIOER LifeECO thermocycler, vortexed again, and centrifuged. Following digestion, 25 µL of neutralization buffer (40 mM Tris, pH 8) was added. DNA samples were stored at − 20 °C until use. Total RNA extraction was performed immediately after gonad sampling using the Monarch RNA extraction kit (New England Biolabs), following the manufacturer’s instructions.

### Sexing and RNA sample selection

Fish were sexed by PCR with gDNA using the genetic sex marker published by Kuhl et al. [24]. Each reaction contained 0.625 µL of 10 µM forward and reverse primers, 20 ng of gDNA, 0.125 µL of NZYTech polymerase (Thermo Fisher Scientific, Waltham, MA, USA), 2.5 µL of 10× NZY Taq buffer, and 3.75 µL of 2 mM dNTP mix (Thermo Fisher Scientific, Waltham, MA, USA), with nuclease-free water added to a final volume of 25 µL. The thermal cycler program was: 95 °C for 3 min; 35 cycles of 95 °C for 30 s, 56 °C for 30 s, and 72 °C for 45 s; followed by a final extension at 72 °C for 8 min. Sex was assessed by analyzing the PCR product on a 2% agarose gel as described by Kuhl et al. [24].

Female and male gonadal RNA samples from genetically sexed fish were identified, and 6 samples of each sex were selected for RNA-Seq analysis. RNA quality was assessed using an Agilent Bioanalyzer 2100. RNA integrity numbers (RIN) between 8 and 10 indicated high-quality RNA in the selected samples (Additional File 2).

### RNA-Seq

All RNA sequencing was performed by Macrogen (Korea). RNA integrity was confirmed using the TapeStation RNA ScreenTape system prior to library construction. Libraries were prepared using the Illumina TruSeq Stranded Total RNA kit with Ribo-Zero Gold. Sequencing was performed on a NovaSeq 6000 platform, generating 100 bp paired-end reads, with approximately 40 million reads per sample, corresponding to roughly 4 Gb of raw data (in FASTQ format).

### Gonad transcriptome assembly and annotation

The transcriptome was assembled using the Siberian sturgeon gonadal transcriptome [20] as a reference. Raw read files were processed with fastp v0.23.4 [25] using default parameters to remove adapters and low-quality bases. Cleaned reads were then aligned with BWA-MEM v0.7.17 [26] against the GICD01.1 Siberian sturgeon reference transcriptome available in the Transcriptome Shotgun Assembly (TSA) database at NCBI. Alignments were sorted, compressed, and indexed with SAMtools v1.x [27] using the sort, view, and index functions with default parameters. Expression levels were quantified with SAMtools idxstats, and expression tables were generated using standard Unix cut and paste commands.

### Differential gene expression analysis

Female and male samples were compared. Each gene was quantified as fragments per kilobase of transcript per million mapped reads (FPKM). Differential expression analysis between the two sexes was performed in R v4.3.1 using the Bioconductor package edgeR. A false discovery rate (FDR) < 0.05 was used as the threshold for defining differentially expressed genes (DEGs). Genes meeting this criterion were considered differentially expressed. The functions calcNormFactors, estimateCommonDisp, estimateTagwiseDisp, and exactTest were applied with default parameters, as described in the edgeR user guide. DEGs overexpressed in females and males were analyzed separately for Gene Ontology (GO) enrichment.

### Identification of protein-coding and non-coding RNA

Female- and male-biased DEG nucleotide sequences were translated using the ExPASy Translate tool (https://web.expasy.org/translate/) to determine which of them encoded proteins. The amino acid sequences of predicted coding ORFs (open reading frames) were compared against public databases using BLASTp (NCBI) to assign gene identities. If no protein-coding ORF was detected, the contig was classified as “non-coding RNA” [28]. Non-coding RNAs were not further analyzed due to the lack of a published Siberian sturgeon genome.

### GO enrichment analysis

(GO) enrichment was performed using topGO [29] (http://bioconductor.org/) with the classic algorithm and Fisher’s exact test. For each list of differentially expressed genes (DEGs), enrichment was assessed for Biological Process (BP), Molecular Function (MF), and Cellular Component (CC) categories.

All DEGs were mapped to GO terms in the Gene Ontology database, and statistical enrichment analysis was performed. GO terms with an adjusted *p* < 0.05 were considered significantly enriched (Additional Files 3 and 4).

### Statistical analysis

The assumptions of normality and homogeneity of variance were evaluated for length and weight data and for expression-level comparisons between clusters. When these assumptions were met, Student’s t-test was applied; if the assumptions were not met, the Mann–Whitney test was used [30]. Statistical significance was set at *p* < 0.05.

## Results

### Differential expression between female and male gonads

No differences in length or weight were detected between males and females used for transcriptomic analysis (t-test, *p* > 0.05).

**Fig. 1:**
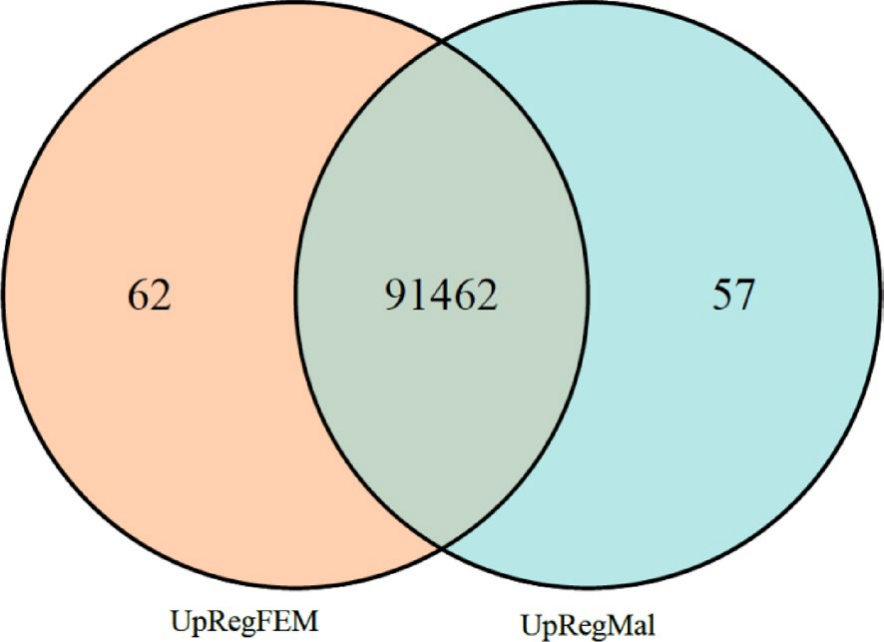
Venn diagram representing the 62 upregulated contigs in females (FDR < 0.05) (UpRegFEM); 57 upregulated contigs in males (FDR < 0.05) (UpRegMal); and 91,462 non-significant contigs (FDR > 0.05)

The assembled gonadal transcriptome of female and male Siberian sturgeon 2.5 months of age produced 91,581 contigs. Of these, 119 were differentially expressed between sexes (FDR < 0.05): 0.07% (*n* = 62) were upregulated in females and 0.06% (*n* = 57) in males (Fig. 1, Additional Files 5 and 6). The remaining 91,462 contigs showed no significant differences. Only 32 female-biased and 29 male-biased contigs encoded proteins (Additional Files 7 and 8). The remaining contigs corresponded to overexpressed non-coding RNA (Additional Files 5 and 6).

### Volcano plot analysis of upregulated sequences

**Fig. 2:**
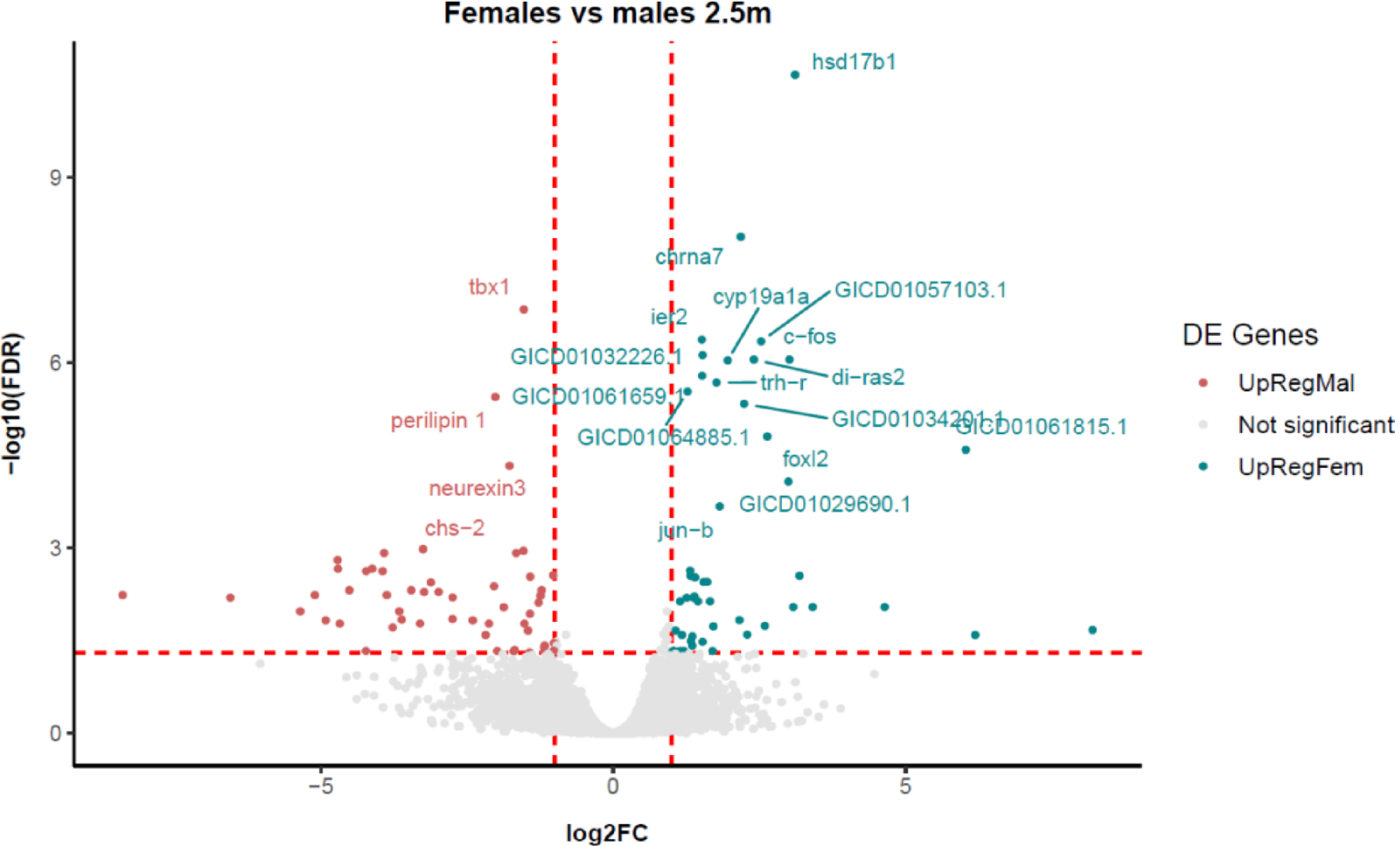
Volcano plot representation of the common contigs (grey) and contigs upregulated in females (turquoise) and males (red). Dotted lines indicate arbitrary thresholds for reference

Figure 2 shows that *hsd17b1* and *tbx1* were the most significantly upregulated genes in females and males, respectively. The results were markedly more significant for *hsd17b1* than for other female genes (FDR = 2.18E^− 11^), such as *cyp19a1* (FDR = 9.17E^− 07^) or *foxl2* (FDR = 1.57E^− 05^). In males, *tbx1* (FDR = 1.38E^− 07^) was most significantly upregulated, followed by *perilipin 1* (*plin1*, 3.59E^− 06^) and *neurexin 3* (*nrxn3*, 4.69E^− 05^).

### Heatmap representation of differentially expressed genes

**Fig. 3:**
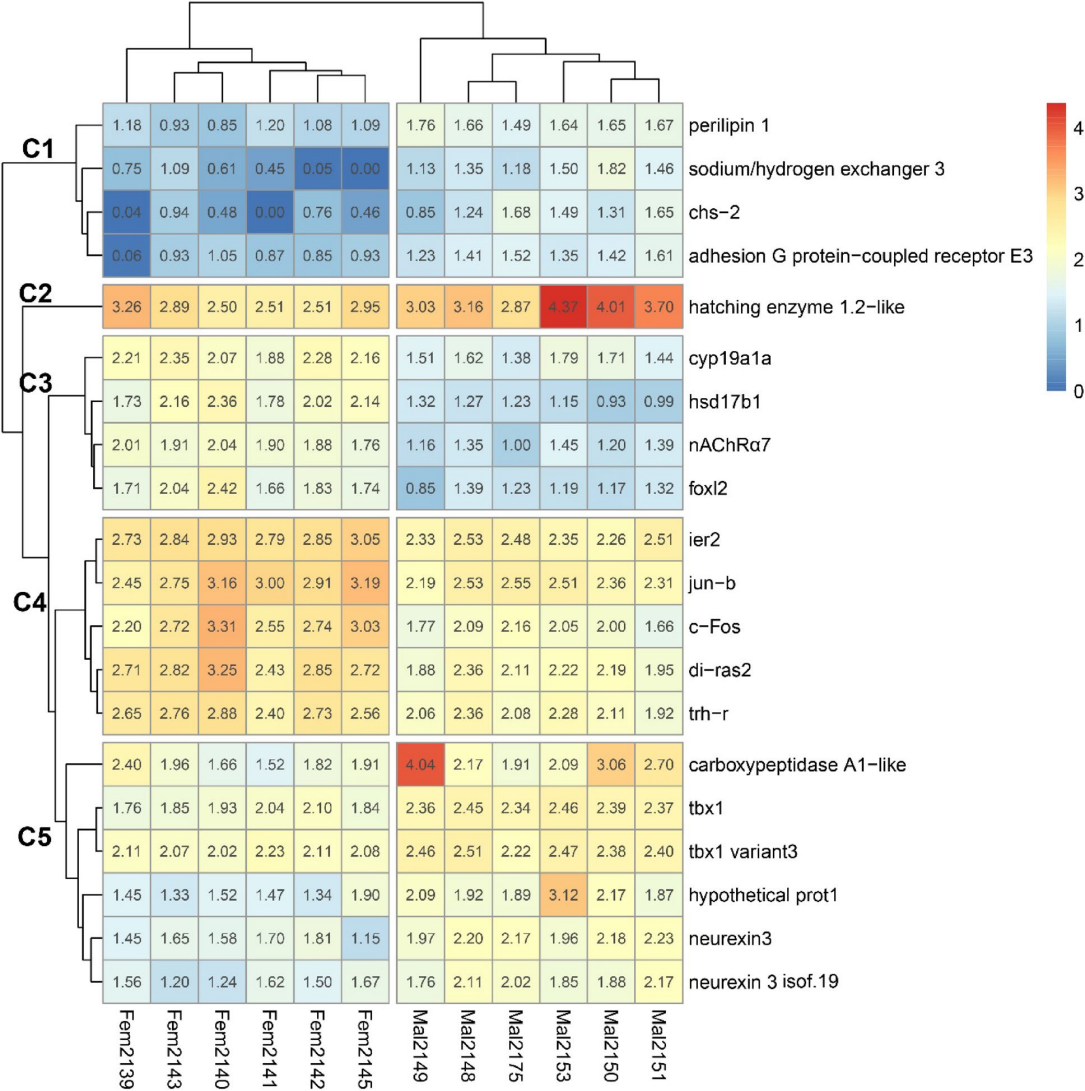
Heatmap of the top 20 codifying contigs. Each line corresponds to one contig and each column to one sample from male (Mal) or female (Fem) groups. The scale from 0 to 4 corresponds to level of contig expression (Z-score). C1 to C5 are the five different clusters

The top 20 coding contigs were separated into five clusters according to expression pattern similarity (Fig. 3). Cluster 1 (C1) included *plin1*, *slc9a3* (sodium/hydrogen exchanger 3), *chs2-like* (chitin synthase 2-like), and *adgre3* (adhesion G protein-coupled receptor E3), all of which were male-biased. Cluster 2 (C2) consisted of a single male-biased gene encoding the hatching enzyme 1.2-like protein.

Clusters 3 (C3) and 4 (C4) contained female-biased genes. C3 grouped together *hsd17b1*, *cyp19a1*, *foxl2*, and *chrna7* (alpha-7 nicotinic cholinergic receptor subunit). C4 included the immediate early response gene *ier2*, transcription factors such as *jun-b* and *c-fos*, signaling factors such as *di-ras2* (Ras-related protein), and hormone-related genes such as *trh-r* (thyrotropin-releasing hormone receptor). Between the female-biased clusters, genes in C4 showed significantly higher expression than those in C3 (Shapiro–Wilk *p* = 0.09; Levene *p* = 0.02; Mann–Whitney *p* = 0.005).

C5 contained the male-biased genes *tbx1* and *nrxn3*, along with one isoform (*neurexin 3 isoform 19*). It also included *carboxypeptidase A1-like* and a hypothetical protein-coding gene with an open reading frame (ORF) that has not yet been characterized in available databases.

In addition to the genes represented in the heatmap, other genes were found to be overexpressed in female gonads. Among them, serotonin N-acetyltransferase (*aanat*), a key enzyme in melatonin biosynthesis, and the one coding for the transcription factor Egr1 (*early growth response 1*) stand out as particularly.

### Gene ontology (GO) enrichment

GO analysis of female-upregulated contigs (*n* = 62) revealed 186 Biological Processes (BP), three Cellular Components (CC), and six Molecular Function (MF) GO terms that were significantly enriched (adjusted *p* < 0.05) (Additional File 3). In males, the Gene Ontology analysis of upregulated contigs (*n* = 57) identified 256 enriched GO terms in the BP category, 7 in CC, and 23 in MF (p adjusted *p* < 0.05) (Additional File 4).

From the list of enriched GO terms, we selected those associated with gonadal processes related to sex differentiation and maturation in females and males (Additional Files 9 and 10). Notable GO terms identified in females included “steroid hormone biosynthesis,” “gonadotropin response” (FSH, LH), “granulosa cell differentiation,” “melatonin metabolism” and “transcription factors such as AP-1” (Additional File 3 and 9). In males, “hormone metabolic process” and “retinoic acid signaling” were among the most relevant differentially enriched GO terms (Additional File 10).

## Discussion

This study presents the first transcriptomic analysis of undifferentiated gonads from genetically sexed Siberian sturgeon during early molecular sex differentiation, at 2.5 months of age [9]. This approach provides a snapshot of this complex process during a critical developmental period, pointing to gene and pathway networks that may drive gonadal differentiation [9, 31]. Gene expression profiles were largely conserved between the sexes, with 99.87% of the studied contigs showing no expression differences between males and females. Early female and male sexual differentiation, therefore, is likely guided by the 0.13% of the total genes expressed in gonads showing sex-biased activation (0.07% in future ovaries, 0.06% in future testis). Most of the genes are likely crucial for the survival and proliferation of gonadal cells, as observed in mammals [11].

### Validation of the gonadal transcriptome

The transcriptome was validated by the identification of known female-specific genes (e.g., *cyp19a1*, *foxl2*, *hsd17b1*) among the top differentially expressed contigs [9, 31]. These results are consistent with our previous qPCR studies with and without sex markers [9, 31–33]. Moreover, the crucial role of estradiol-17β in Siberian sturgeon ovarian differentiation has been reported during phenotypic male-to-female sex reversal treatments with exogenous E2 [10]. These validations based on previous work [9, 10] strengthens these new results and supports the involvement of the newly identified overexpressed genes in female and male sexual differentiation pathways, including their involvement from early stages in this complex process.

### Novel candidates in sex differentiation

New gene candidates for sex differentiation emerged from the heatmap clustering analysis. Estrogens-related genes (*hsd17b1*, *cyp19a1*, *foxl2*) were grouped in Cluster 3, while Cluster 4 included other genes overexpressed in female gonads such as *jun-b*, *c-fos*, *ier2*, *di-ras2*, and *trh-r*. The uniform expression levels within each cluster suggest the presence of synexpression groups of genes that could be co-expressed and potentially co-regulated, indicating that these genes may function together within common regulatory pathways to reach a common goal, in this case to drive sex-specific development.

Many of the aforementioned genes may be estrogens-responsive or act downstream of estrogen signaling. Immediate early genes such as *jun-b* and *c-fos* are well known to be rapidly stimulated by estrogens [34, 35]. Although not included in the heatmap, *egr1* was significantly upregulated in females in our differential expression analysis. *egr1* is expressed in somatic cells of the ovary and testis and can be stimulated by 17β-estradiol [36–38]. This gene functions as a transcription factor regulating cellular growth and differentiation in response to mitogenic and stress signals [39–41]. In *Acanthopagrus latus*, a hermaphroditic fish, *egr1* is overexpressed in the female pituitary during the transition from an ovotestis to a definitive ovary, consistent with a role in female differentiation [42]. In mammals, *Egr1* expression varies with the estrous cycle, and its overexpression influences brain plasticity and female-specific behaviors [43], further supporting its importance in female-related pathways.

Although little is known about their regulation during ovarian differentiation, the expression of *ier2* has been shown to be stimulated by E2 in the rat uterus [44], and the presence of *di-ras2* in Cluster 4 suggests that this gene could also be modulated by estrogens in the gonadal differentiation context.

Finally, *trh-r* also appears to be estrogen-sensitive, as estrogens upregulate TRH receptor expression in mammotropic cells [45–47]. Thus, we suggest that genes in Cluster 4 are part of an estrogens-driven synexpression group, in which functionally related genes are coordinately expressed in response to estrogens produced by genes in Cluster 3 (*hsd17b1*, *cyp19a1*, *foxl2*).

### Functional overview of the genes in cluster 4

*jun-b* and *c-fos* emerge as particularly relevant due to their established roles as immediate early response genes [48, 49]. These genes encode transcription factors that dimerize to form the AP-1 complex, which can regulate downstream targets including *cyp19a1* [50, 51], thereby indirectly influencing estrogens production. The AP-1 complex, as well as *jun* and *fos* individually, are broadly involved in cell proliferation and differentiation [49, 52–54].

Evidence from other species supports their potential role in ovarian development: *c-fos* has been implicated in ovary formation in *Drosophila* [55], while in chickens, silencing of *jun* causes phenotypic masculinization by repressing the classical female genes *foxl2*,* cyp19a1*, and *esr1* and upregulating male pathway genes such as *dmrt1* and *sox9* [56]. To date, neither *jun-b* nor *c-fos* have been reported in the context of gonadal differentiation in fish; nevertheless, our findings suggest that they may play key regulatory roles in early ovarian differentiation in Siberian sturgeon.

*ier2* encodes a transcription factor that belongs to the *immediate early gene* group. This gene is a downstream target of fibroblast growth factor (FGF) signaling that plays a role in the establishment of left-right asymmetry and convergent extension movements in zebrafish [57] and Xenopus [58]. It is known that FGF signaling acts on multiple types of somatic cells within the developing gonad in the larval and pupal stages of Drosophila and is necessary for maintaining fertility [59]. In mammals *Ier2* expression has been detected in the ovary [60] and, more specifically, in the rat uterus, Arao et al. [44] demonstrated that the gene is stimulated by 17β-estradiol (E2).

*di-ras2* acts as a tumor suppressor by inhibiting the Wnt/β-catenin pathway [61, 62], which regulates expression of genes involved in cell proliferation, differentiation, and maintenance across species and plays a well-established role in gonadal development in mammals [11]. Although *di-ras2* has not been directly associated with gonadal differentiation, we hypothesize that it may influence the proliferation of specific cell types stimulated by estrogens. This effect could be mediated through pathways regulated by immediate early genes such as *jun-b* and *c-fos*, which can act individually or as AP-1 dimers.

Activation of *trh-r* in female gonads contrasts with the typical association of the thyroid axis with male differentiation [63]. In *Xenopus laevis* larvae, inhibition of TSH leads to feminization [64, 65], while in zebrafish, administration of thyroid hormones promotes testis development by upregulating male-associated and downregulating female-associated genes [66]. In medaka (*Oryzias latipes*) and rice field eel (*Monopterus albus*), thyroid hormone treatment likewise activates the male differentiation pathway and stimulates the production of 11-oxygenated androgens [67, 68].

These findings suggest that the thyroid axis may function differently in Siberian sturgeon, possibly in a species-specific manner. One possibility is that TRH is produced by the ovary itself and acts in an autocrine or paracrine fashion to regulate female gonadal development. However, our previous tissue and gonadal transcriptome analyses [20, 69] showed that *trh* is highly expressed in the brain but not in the gonads of either sex at any developmental stage. This raises the question of whether hypotalamic-produced TRH circulates to act on gonadal *trh-r* at 2.5 months of age, or whether TRH receptors instead interact with another locally produced factor that activates the female pathway.

Interestingly, the female-biased genes in Cluster 4, which have received less attention in the literature in the context of sex differentiation, showed significantly higher expression than those in Cluster 3, which contains the classic estrogens-production genes (*hsd17b1*,* cyp19a1*, and *foxl2*). This finding highlights their potential importance in the early development and regulation of female gonads and suggests that these two gene clusters may interact during the initial stages of gonadal differentiation.

### Novel hormonal players in ovarian differentiation

The discovery of serotonin *N*-acetyltransferase overexpression and enrichment of the GO term “melatonin metabolism” is particularly novel in the context of ovarian sex differentiation. Melatonin has traditionally been linked to circadian rhythms and reproductive processes, and it is produced in both male and female gonadal tissues [70, 71]. In *Cyprinus carpio*, treatment with aromatase inhibitors during early sex differentiation to induce female-to-male transdifferentiation is accompanied by reduced estradiol and melatonin levels, suggesting a cooperative role of these hormones in ovarian differentiation [72]. These findings raise important questions about whether melatonin acts synergistically with estradiol in promoting female development, or whether it can influence ovarian differentiation independently. Further hormone administration studies will be required to clarify this point.

### Ovarian regulatory model near the onset of sex differentiation

As we previously demonstrated [9, 10], future ovaries are capable of producing estrogens by 2.5 months of age. This capacity is supported by activation of the enzymes *hsd17b1* and *cyp19a1* (aromatase), which are essential for estrogens biosynthesis, and of the transcription factor *foxl2*, which stimulates aromatase expression.

**Fig. 4:**
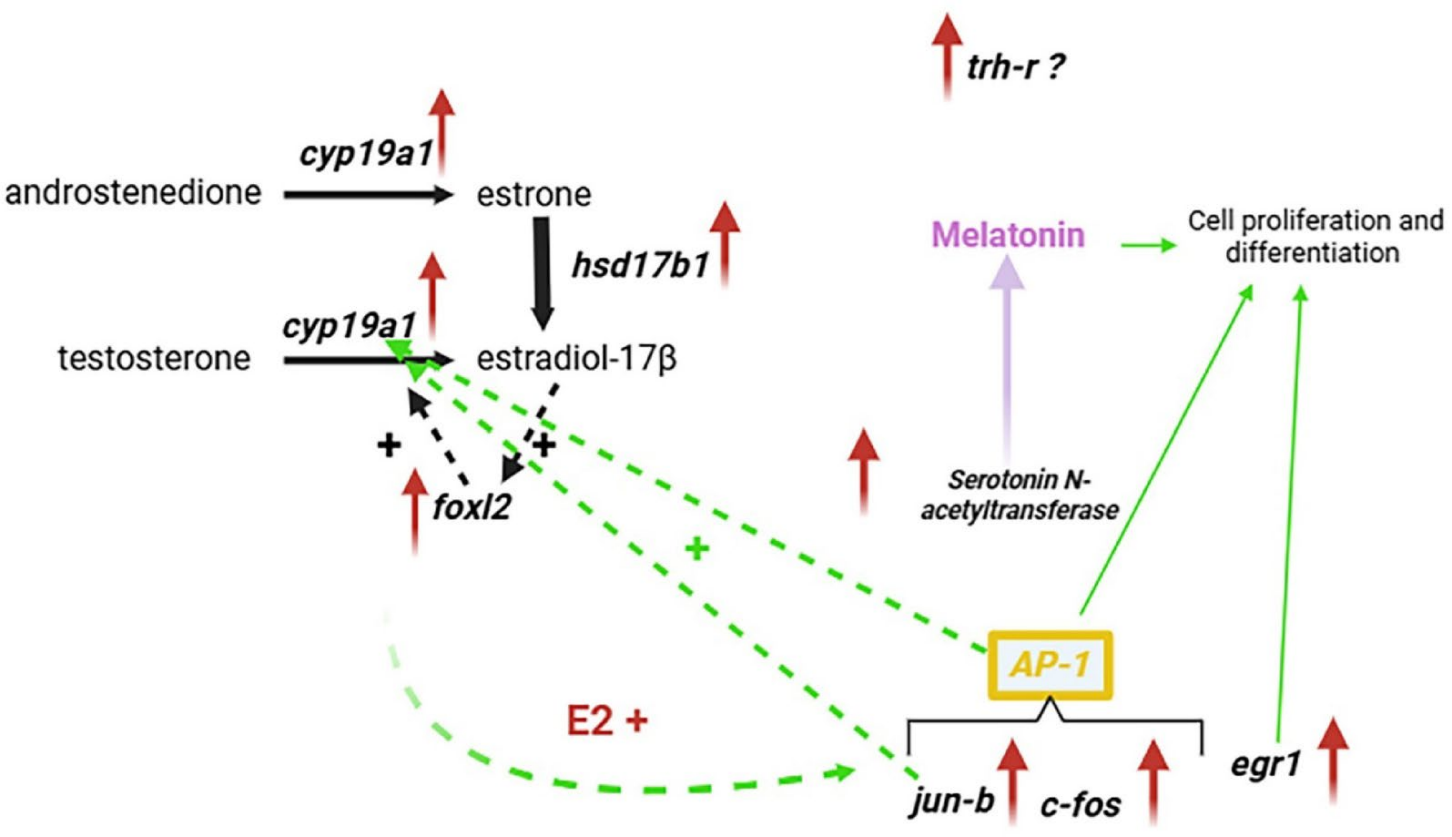
Proposed regulatory model for early ovarian sex differentiation in Siberian sturgeon. Red arrows indicate upregulated genes. Thick solid black arrows represent well-characterized regulatory pathways. Dashed black arrows indicate known positive regulatory interactions. Thin solid green arrows denote established functions involving factors activated in this study. Dashed green arrows represent potential positive regulatory interactions among components. Purple highlights the proposed production of melatonin. A question mark (?) indicates the uncertain role of *trh-r*

The present study confirms the early estrogen production pathway and identifies new estrogens targets, such as immediate early genes *jun-b*, *c-fos*, and *egr-1* (Fig. 4). *c-fos*, *jun-b*, and *egr-1* are activated downstream, stimulated by estrogen signaling. *jun-b* can stimulate aromatase either independently or by forming a dimer with *c-fos*, which acts as a transcription factor called AP1. We propose a new positive feedback loop for aromatase stimulation through *jun-b* and AP1, independent of *foxl2* (Fig. 4, dashed green arrows).

In turn, AP-1, formed by the products of *jun-b* and *c-fos*, together with *egr1*, may regulate the cell proliferation and differentiation processes required for development of the female gonad (Fig. 4, solid green arrows). This proposed network consolidates the estrogenic identity of the ovary near the onset of molecular sexual differentiation, with a gene node downstream of E2 capable of two major functions: maintaining ovarian estrogens levels through stimulation of aromatase, and regulating cell proliferation and differentiation.

Moreover, the potential involvement of hormones such as melatonin and TRH, which may act synergistically with estrogens (Fig. 4), adds further complexity to early ovarian differentiation in fish and other vertebrates. This underscores the importance of newly identified sex-differentiation-related genes in the earliest stages of ovarian development.

#### Potential role of non-coding RNAs in gonadal differentiation

The overexpression of non-coding transcripts in both male and female gonads suggests the involvement of additional regulatory pathways of gene transcription. In fish, long non-coding RNAs can directly interact with chromatin and regulate transcription through epigenetic modifications [73]. Epigenetic regulation of sex-differentiation genes has been reported in *Cynoglossus semilaevis* and linked to sexual differentiation [74, 75]. It is therefore possible that some of the non-coding transcripts identified here act as epigenetic factors influencing gene transcription during early sex differentiation in *Acipenser baerii*. However, without a reference genome, we cannot definitively classify these contigs as long non-coding RNAs, and such analyses will require new genome assemblies. Nevertheless, this represents a promising direction for future genomic and epigenetic research in sturgeons.

### Perspectives on testicular differentiation: early candidates in a delayed process

The early male differentiation pathway remains poorly understood. Here, 29 contigs with coding ORFs were overexpressed in males. Unlike the ovarian molecular signature, male gonads at this early timepoint lacked the classical genes typically involved in testicular differentiation, consistent with observations at more advanced stages in previous studies [9, 31]. Notably, this is the first report of *tbx1*, a gene previously linked to testicular differentiation in trout, being overexpressed at this early stage of development [76, 77]. Moreover, *tbx1* was the most strongly expressed of all male-biased genes, representing a promising early marker of testicular differentiation.

*tbx1* also has well-established roles in male gonadal fate across vertebrates. Functional evidence from amphibians and reptiles [78–80] shows that *tbx1* represses the classical ovarian pathway (e.g., *cyp19a1*,* foxl2*) while stimulating testicular development (e.g., *amh*,* dmrt1*,* wt1*,* cyp26a*). In *Andrias davidianus*, feminizing estradiol treatments repress *tbx1* expression [78]. Interestingly, at this early stage of development, no genes related to androgen production were detected in future testes, suggesting that these factors are not directly involved in early male gonadal differentiation, an idea previously proposed by Lasalle et al. [9]. Taken together, these findings indicate that in an estrogen-free microenvironment that creates the conditions for male differentiation, *tbx1* may act as an early upstream regulator of testicular development in *A. baerii*.

*mmp9*, another important male upregulated gene, encodes a metalloproteinase involved in extracellular matrix remodeling, a process critical for testis formation [81] and germ cell migration [82–84]. In sturgeon, metalloproteinases have previously been linked to gonadal development [31]. A similar role has been described in the protogynous species *Synbranchus marmoratus*, where metalloproteinases facilitate ovarian regression and testicular development [85]. Together, the available evidence suggests that *mmp9* may play a structural and organizational role in testicular morphogenesis in sturgeon, enabling germ cell migration during the early stages of testicular differentiation.

Other significantly male-skewed genes remain functionally uncharacterized, possibly reflecting unknown sex-specific functions before or during testicular differentiation. Studying these factors will be essential for completing our understanding of the male differentiation network.

### Sex-specific GO term enrichment

The functional pathways derived from the GO analysis reflect the progression of sex differentiation in males and females. The two sexes showed distinct patterns of enriched terms. The differentially activated GO terms in females were closely associated with sex differentiation, determination, and hormone production (i.e. estrogens), along with metabolic processes in the gonads. In contrast, fewer of the differentially expressed GO terms in males appeared to be linked to sex-specific processes, and instead were mainly associated with retinoic acid and general hormone metabolism. None of the male-biased GO terms were related to androgen production or receptivity [86, 87]. In females, the abundance of GO terms directly associated with gonadal processes indicates a more advanced gonadal development at this stage compared to the male process. The pattern of male and female GO terms suggests that sex differentiation progresses more slowly in males than females, consistent with results in Siberian sturgeon in later stages of gonad development [31].

## Conclusion

Our results support the idea that molecular sexual differentiation begins by 2.5 months of age and is more advanced in females than males at this stage.

This first transcriptomic analysis of undifferentiated gonads reveals the activation of both classic estrogens-production genes and novel female-biased candidates, including immediate early response genes as well as genes not previously linked to estrogen signaling. The established male factor *tbx1* was overexpressed in males, along with previously unrecognized candidates. Furthermore, as in prior studies, no sex-biased genes related to androgen production were detected, suggesting that early male gonadal development may proceed independently of steroid production.

Overall, this exploratory study highlights the complexity of pathways involved in gonadal differentiation and proposes new hypotheses regarding the mechanisms and factors that may operate during the early stages of gonadal development in basal fish.

## Supplementary Information

Supplementary Material 1

Supplementary Material 2

Supplementary Material 3

Supplementary Material 4

Supplementary Material 5

Supplementary Material 6

Supplementary Material 7

Supplementary Material 8

Supplementary Material 9

Supplementary Material 10

## Data Availability

The datasets generated and/or analyzed during the current study are available in the NCBI repository, under the BioProject PRJNA589957 ( https://www.ncbi.nlm.nih.gov/search/all/?term=PRJNA589957 ) published in Vizziano-Cantonnet et al. (2018). The corresponding GICD reference for each sequence can be found under the “nucleotide” category in NCBI. Sequencing counts data generated during the current study are not publicly available as they are confidential laboratory data but are available from the corresponding author on reasonable request.

## References

[1] Gamble T, Zarkower D. Sex determination. Curr Biol. 2012;22:257–62. 10.1016/j.cub.2012.02.054 PMC11003734 22537624

[2] Penman DJ, Piferrer F. Fish gonadogenesis. Part I: genetic and environmental mechanisms of sex determination. Rev Fish Sci. 2008;16:16–34.

[3] Guiguen Y, Fostier A, Piferrer F, Chang CF. Ovarian aromatase and estrogens: a pivotal role for gonadal sex differentiation and sex change in fish. Gen Comp Endocrinol. 2010;165:352–66. 19289125 10.1016/j.ygcen.2009.03.002

[4] Kitano J, Ansai S, Takehana Y, Yamamoto Y. Diversity and convergence of sex-determination mechanisms in teleost fish. Annul Rev Anim Biosci. 2024;12:233–59. 37863090 10.1146/annurev-animal-021122-113935

[5] Nagahama Y, Chakraborty T, Paul-Prasanth B, Ohta K, Nakamura M. Sex determination, gonadal sex differentiation, and plasticity in vertebrate species. Physiol Rev. 2021;101:1237–308. 33180655 10.1152/physrev.00044.2019

[6] Piferrer F, Guiguen Y. Fish gonadogenesis. Part II: molecular biology and genomics of sex differentiation. Rev Fish Sci. 2008;16:35–55.

[7] Yamamoto Y, Luckenbach JA. Sex determination and gonadal sex differentiation. 2024.

[8] Yamamoto T-O. Sex differentiation. Fish physiol. 1969;3:117 – 75.

[9] Lasalle A, Benech-Correa G, Brunet FG, Vizziano‐Cantonnet D. *hsd17b1* is a key gene for ovarian differentiation of the Siberian sturgeon. Mol Reprod Dev. 2024;91:e23729. 38282315 10.1002/mrd.23729

[10] Vizziano-Cantonnet D, Benech-Correa G, Lasalle-Gerla A. Estradiol-17β but not 11β-hydroxyandrostenedione induces sex transdifferentiation in Siberian sturgeon. Aquaculture. 2025;596:741735.

[11] Brennan J, Capel B. One tissue, two fates: molecular genetic events that underlie testis versus ovary development. Nat Rev Genet. 2004;5:509–21. 15211353 10.1038/nrg1381

[12] Qi Q, Dong Z, Zhang N, Wang L, Shao C, Xu W. Cloning, expression and functional analysis of the desert Hedgehog (*dhh*) gene in Chinese tongue sole (*Cynoglossus semilaevis*). Gene Expr Patterns. 2021;39:119163. 33359643 10.1016/j.gep.2020.119163

[13] Crespo B, Gomez A, Mazón MJ, Carrillo M, Zanuy S. Isolation and characterization of Ff1 and Gsdf family genes in European sea bass and identification of early gonadal markers of precocious puberty in males. Gen Comp Endocrinol. 2013;191:155–67. 23791759 10.1016/j.ygcen.2013.06.010

[14] Gautier A, Le Gac F, Lareyre J-J. The *Gsdf* gene locus harbors evolutionary conserved and clustered genes preferentially expressed in fish previtellogenic oocytes. Gene. 2011;472:7–17. 21047546 10.1016/j.gene.2010.10.014

[15] Lareyre J-J, Ricordel M-J, Mahé S, Goupil A-S, Vizziano D, Bobe J, et al. Two new TGF beta members are restricted to the gonad and differentially expressed during sex differentiation and gametogenesis in trout. Cybium. 2008;32(Suppl):202.

[16] Sawatari E, Shikina S, Takeuchi T, Yoshizaki G. A novel transforming growth factor-β superfamily member expressed in gonadal somatic cells enhances primordial germ cell and spermatogonial proliferation in rainbow trout (*Oncorhynchus mykiss*). Dev Biol. 2007;301:266–75. 17109839 10.1016/j.ydbio.2006.10.001

[17] Hsu C-w, Chung B-c. Evolution, expression, and function of gonadal somatic cell-derived factor. Front Cell Dev Biol. 2021;9:684352. 34307362 10.3389/fcell.2021.684352 PMC8292791

[18] Kaneko H, Ijiri S, Kobayashi T, Izumi H, Kuramochi Y, Wang D-S, et al. Gonadal soma-derived factor (*gsdf*), a TGF-beta superfamily gene, induces testis differentiation in the teleost fish *Oreochromis niloticus*. Mol Cell Endocrinol. 2015;415:87–99. 26265450 10.1016/j.mce.2015.08.008

[19] Zhu Y, Meng L, Xu W, Cui Z, Zhang N, Guo H, et al. The autosomal *Gsdf* gene plays a role in male gonad development in Chinese tongue sole (*Cynoglossus semilaevis*). Sci Rep. 2018;8:17716. 30531973 10.1038/s41598-018-35553-7 PMC6286346

[20] Klopp C, Lasalle A, Di Landro S, Vizziano-Cantonnet D. RNA-Seq transcriptome data of undifferentiated and differentiated gonads of Siberian sturgeon. Data Brief. 2020;31:105741. 10.1016/j.dib.2020.105741 PMC7280750 32529009

[21] Benech-Correa G, Lasalle Gerla AE, Vizziano Cantonnet D. *gsdf* is the only major pro-male gene activated during the molecular sex differentiation period of Siberian sturgeon. 30th CECE & 9th ISFE Joint Conference of the European Society for Comparative Endocrinology and of the International Society for Fish Endocrinology; Faro, Portugal. 2022.

[22] Ruban GI. Geographical Distribution, ecological and biological characteristics of the Siberian sturgeon species. In: Williot P, Nonnotte G, Vizziano-Cantonnet D, Chebanov M, editors. The Siberian sturgeon (*Acipenser baerii*, Brandt, 1869) volume 1 - Biology. Cham: Springer; 2018. pp. 3–28.

[23] Rzepkowska M, Ostaszewska T. Proliferating cell nuclear antigen and Vasa protein expression during gonadal development and sexual differentiation in cultured Siberian (*Acipenser baerii* Brandt, 1869) and Russian (*Acipenser gueldenstaedtii* Brandt & Ratzeburg, 1833) sturgeon. R Rev Aquacult. 2013;6:75–88.

[24] Kuhl H, Guiguen Y, Höhne C, Kreuz E, Du K, Klopp C, et al. A 180 Myr-old female-specific genome region in sturgeon reveals the oldest known vertebrate sex determining system with undifferentiated sex chromosomes. Philos T R Soc B. 2021;376:20200089. 10.1098/rstb.2020.0089 PMC8273502 34247507

[25] Chen S, Zhou Y, Chen Y, Gu J. Fastp: an ultra-fast all-in-one FASTQ preprocessor. Bioinformatics. 2018;34:i884–90. 30423086 10.1093/bioinformatics/bty560 PMC6129281

[26] Li H, Durbin R. Fast and accurate short read alignment with Burrows–Wheeler transform. Bioinformatics. 2009;25:1754–60. 19451168 10.1093/bioinformatics/btp324 PMC2705234

[27] Li H, Handsaker B, Wysoker A, Fennell T, Ruan J, Homer N, et al. The sequence alignment/map format and samtools. Bioinformatics. 2009;25:2078–9. 19505943 10.1093/bioinformatics/btp352 PMC2723002

[28] Palazzo AF, Lee ES. Non-coding RNA: what is functional and what is junk? Front Genet. 2015;6:2. 25674102 10.3389/fgene.2015.00002 PMC4306305

[29] Alexa A, topGO. Enrichment Analysis for Gene Ontology. R package version. 2016;2(0).

[30] Sokal RR, Rohlf FJ. Biometry: the principles and practice of statistics in biological research. In: WH Freeman editor. New York, USA; 1995.

[31] Vizziano-Cantonnet D, Lasalle A, Di Landro S, Klopp C, Genthon C. De Novo transcriptome analysis to search for sex-differentiation genes in the Siberian sturgeon. Gen Comp Endocrinol. 2018;268:96–109. 30081002 10.1016/j.ygcen.2018.08.007

[32] Lasalle A, Norbis W, Vizziano-Cantonnet D. Sex identification of morphologically‐undifferentiated Siberian sturgeon with statistical analysis of gene expression patterns. J Appl Ichthyol. 2021;37:835–46.

[33] Vizziano-Cantonnet D, Di Landro S, Lasalle A, Martínez A, Mazzoni TS, Quagio‐Grassiotto I. Identification of the molecular sex‐differentiation period in the Siberian sturgeon. Mol Reprod Dev. 2016;83:19–36. 26461178 10.1002/mrd.22589

[34] Fujimoto J, Hori M, Ichigo S, Morishita S, Tamaya T. Estrogen induces expression of *c-fos* and *c-jun* via activation of protein kinase C in an endometrial cancer cell line and fibroblasts derived from human uterine endometrium. Gynecol Endocrinol. 1996;10:109 – 18. 10.3109/09513599609097900 8701784

[35] Webb DK, Moulton BC, Khan SA. Estrogen induces expression of *c-jun* and *jun-b* protooncogenes in specific rat uterine cells. Endocrinology. 1993;133:20 – 8. 10.1210/endo.133.1.8319568 8319568

[36] Kim H-R, Kim YS, Yoon JA, Lyu SW, Shin H, Lim HJ, et al. Egr1 is rapidly and transiently induced by Estrogen and bisphenol A via activation of nuclear Estrogen receptor-dependent ERK1/2 pathway in the uterus. Reprod Toxicol. 2014;50:60–7. 25461906 10.1016/j.reprotox.2014.10.010

[37] Punetha M, Kumar S, Paul A, Jose B, Bharati J, Sonwane A, et al. Deciphering the functional role of EGR1 in prostaglandin F2 alpha induced luteal regression applying CRISPR in corpus luteum of Buffalo. Biol Res. 2021;54:9. 10.1186/s40659-021-00333-7 PMC7953609 33712084

[38] Russell DL, Doyle KMH, Gonzales-Robayna I, Pipaon C, Richards JS. Egr-1 induction in rat granulosa cells by follicle-stimulating hormone and luteinizing hormone: combinatorial regulation by transcription factors Cyclic adenosine 3′, 5′-monophosphate regulatory element binding protein, serum response factor, sp1, and early growth response factor-1. Mol Endocrinol. 2003;17:520–33. 12554779 10.1210/me.2002-0066

[39] Bae M-H, Jeong C-H, Kim S-H, Bae M-K, Jeong J-W, Ahn M-Y, et al. Regulation of Egr-1 by association with the proteasome component C8. BBA-Mol Cell Res. 2002;1592:163–7. 10.1016/s0167-4889(02)00310-5 12379479

[40] Gashler A, Sukhatme VP. Early growth response protein 1 (Egr-1): prototype of a zinc-finger family of transcription factors. Prog Nucleic Acid Res. 1995;50:191–224. 10.1016/s0079-6603(08)60815-6 7754034

[41] Liu J, Grogan L, Nau MM, Allegra CJ, Chu E, Wright JJ. Physical interaction between p53 and primary response gene Egr-1. Int J Oncol. 2001;18:863–70. 11251186 10.3892/ijo.18.4.863

[42] Zhang J, Yang J, Ma Z, Pu H, Zhang T, Guo J, et al. Molecular and cellular regulation on sexual fate and gonadal development in hermaphrodite Yellowfin seabream *Acanthopagrus latus*. Aquaculture Rep. 2024;34:101913.

[43] Rocks D, Purisic E, Gallo EF, Greally JM, Suzuki M, Kundakovic M. Egr1 is a sex-specific regulator of neuronal chromatin, synaptic plasticity, and behaviour. BioRxiv. 2023. 10.1101/2023.12.20.572697. 41390827 10.1038/s41467-025-66217-6 PMC12738549

[44] Arao Y, Kikuchi A, Kishida M, Yonekura M, Inoue A, Yasuda S, et al. Stability of A + U-rich element binding factor 1 (AUF1)-binding messenger ribonucleic acid correlates with the subcellular relocalization of AUF1 in the rat uterus upon Estrogen treatment. Mol Endocrinol. 2004;18:2255–67. 15192077 10.1210/me.2004-0103

[45] De Lean A, Ferland L, Drouin J, Kelly PA, Labrie F. Modulation of pituitary Thyrotropin releasing hormone receptor levels by estrogens and thyroid hormones. Endocrinology. 1977;100:1496–504. 192537 10.1210/endo-100-6-1496

[46] Gershengorn MC, Marcus-Samuels BE, Geras E. Estrogens increase the number of thyrotropin-releasing hormone receptors on mammotropic cells in culture. Endocrinology. 1979;105:171–6. 221199 10.1210/endo-105-1-171

[47] Kimura N, Arai K, Sahara Y, Suzuki H, Kimura N. Estradiol transcriptionally and posttranscriptionally up-regulates thyrotropin-releasing hormone receptor messenger ribonucleic acid in rat pituitary cells. Endocrinology. 1994;134:432–40. 8275956 10.1210/endo.134.1.8275956

[48] Healy S, Khan P, Davie JR. Immediate early response genes and cell transformation. Pharmacol Therapeut. 2013;137:64–77. 10.1016/j.pharmthera.2012.09.001 22983151

[49] O’Donnell A, Odrowaz Z, Sharrocks AD. Immediate-early gene activation by the MAPK pathways: what do and don’t we know? Biochem Soc T. 2012;40:58–66. 10.1042/BST20110636 22260666

[50] Bulun SE, Lin Z, Zhao H, Lu M, Amin S, Reierstad S, et al. Regulation of aromatase expression in breast cancer tissue. Ann NY Acad Sci. 2009;1155:121–31. 19250199 10.1111/j.1749-6632.2009.03705.x

[51] Chen D, Reierstad S, Fang F, Bulun SE. JunD and JunB integrate prostaglandin E2 activation of breast cancer-associated proximal aromatase promoters. Mol Endocrinol. 2011;25(5):767–75. 21393445 10.1210/me.2010-0368 PMC3082330

[52] Angel P, Karin M. The role of Jun, Fos and the AP-1 complex in cell-proliferation and transformation. BBA-Rev Cancer. 1991;1072:129–57. 10.1016/0304-419x(91)90011-9 1751545

[53] van der Burg B, van Selm-Miltenburg AJP, de Laat SW, van Zoelen EJJ. Direct effects of Estrogen on c-fos and c-myc protooncogene expression and cellular proliferation in human breast cancer cells. Mol Cell Endocrinol. 1989;64:223–8. 2507374 10.1016/0303-7207(89)90149-4

[54] Wagner EF. Functions of AP1 (Fos/Jun) in bone development. Ann Rheum Dis. 2002;61(Suppl2):40–2. 10.1136/ard.61.suppl_2.ii40 PMC1766713 12379619

[55] Klein JD, Qu C, Yang X, Fan Y, Tang C, Peng JC. c-Fos repression by Piwi regulates *Drosophila* ovarian germline formation and tissue morphogenesis. PLoS Genet. 2016;12:e1006281. 27622269 10.1371/journal.pgen.1006281 PMC5021354

[56] Zhang M, Xu P, Sun X, Zhang C, Shi X, Li J, et al. *JUN* promotes chicken female differentiation by inhibiting *Smad2*. Cytotechnology. 2021;73:101–13. 33505118 10.1007/s10616-020-00447-y PMC7817717

[57] Hong S-K, Dawid IB. FGF-dependent left–right asymmetry patterning in zebrafish is mediated by Ier2 and Fibp1. Proc Natl Acad Sci USA. 2009;106:2230–5. 19164561 10.1073/pnas.0812880106 PMC2650137

[58] Hong S-K, Tanegashima K, Dawid IB. *XIer2* is required for convergent extension movements during xenopus development. Int J Dev Biol. 2011;55:917. 22252488 10.1387/ijdb.113288sh PMC3261581

[59] Irizarry J, Stathopoulos A. FGF signaling supports *Drosophila* fertility by regulating development of ovarian muscle tissues. Dev Biol. 2015;404:1–13. 25958090 10.1016/j.ydbio.2015.04.023 PMC4469552

[60] Jayasimha C. Expression and hormonal regulation of transcriptional regulatory genes modulated by luteinizing hormone in Buffalo ovarian cells identified using comparative genomics. 2011. (Doctoral dissertation, NDRI).

[61] Shipitsin M, Campbell LL, Argani P, Weremowicz S, Bloushtain-Qimron N, Yao J, et al. Molecular definition of breast tumor heterogeneity. Cancer Cell. 2007;11:259–73. 17349583 10.1016/j.ccr.2007.01.013

[62] Xu X, Zhou W, Chen Y, Wu K, Wang H, Zhang J, et al. Immediate early response protein 2 promotes the migration and invasion of hepatocellular carcinoma cells via regulating the activity of Rho GTPases. Neoplasma. 2020;67:614–22. 32009420 10.4149/neo_2020_190818N781

[63] Tovo-Neto A, da Silva Rodrigues M, Habibi HR, Nóbrega RH. Thyroid hormone actions on male reproductive system of teleost fish. Gen Comp Endocrinol. 2018;265:230–6. 29678724 10.1016/j.ygcen.2018.04.023

[64] Goleman WL, Carr JA, Anderson TA. Environmentally relevant concentrations of ammonium perchlorate inhibit thyroid function and alter sex ratios in developing *Xenopus laevis*. Environ Toxicol Chem. 2002;21:590–7. 11878472

[65] Hayes TB. Steroids as potential modulators of thyroid hormone activity in Anuran metamorphosis. Am Zool. 1997;37:185–94.

[66] Sharma P, Tang S, Mayer GD, Patiño R. Effects of thyroid endocrine manipulation on sex-related gene expression and population sex ratios in zebrafish. Gen Comp Endocrinol. 2016;235:38–47. 27255368 10.1016/j.ygcen.2016.05.028

[67] Castañeda-Cortés DC, Rosa IF, Boan AF, Marrone D, Pagliaro N, Oliveira MA, et al. Thyroid axis participates in high-temperature-induced male sex reversal through its activation by the stress response. Cell Mol Life Sci. 2023;80:253. 37589787 10.1007/s00018-023-04913-6 PMC11071808

[68] Feng K, Su J, Wu Z, Su S, Yao W. Molecular cloning and expression analysis of Thyrotropin-Releasing Hormone, and its possible role in gonadal differentiation in rice field eel *Monopterus albus*. Animals. 2022;12:1691. 10.3390/ani12131691 PMC9264984 35804589

[69] Klopp C, Cabau C, Greif G, Lasalle A, Di Landro S, Vizziano-Cantonnet DJD. Siberian sturgeon multi-tissue reference transcriptome database. Database. 2020;2020. 10.1093/database/baaa082 PMC7687680 33238003

[70] Itoh MT, Ishizuka B, Kuribayashi Y, Amemiya A, Sumi Y. Melatonin, its precursors, and synthesizing enzyme activities in the human ovary. Mol Hum Reprod. 1999;5:402–8. 10338362 10.1093/molehr/5.5.402

[71] Tijmes M, Pedraza R, Valladares L. Melatonin in the rat testis: evidence for local synthesis. Steroids. 1996;61:65–8. 8750434 10.1016/0039-128x(95)00197-x

[72] Singh AK, Singh R. In vivo response of melatonin, gonadal activity and biochemical changes during CYP19 inhibited sex reversal in common carp *Cyprinus Carpio* (L). Anim Reprod Sci. 2013;136:317–25. 23218911 10.1016/j.anireprosci.2012.11.004

[73] Piferrer F. Epigenetics of sex determination and differentiation in fish. In: Han-Ping Wang, Francesc Piferrer, Song-Lin Chen, Zhi-Gang, Shen, editors. Sex control in aquaculture. 2018:65–83.

[74] Liu J, Liu X, Jin C, Du X, He Y, Zhang Q. Transcriptome profiling insights the feature of sex reversal induced by high temperature in tongue sole *Cynoglossus semilaevis*. Front Genet. 2019;10:522. 31191622 10.3389/fgene.2019.00522 PMC6548826

[75] Zhang Y, Xu F, Li H, Chen S. Differential methylation reveals pathways associated with sex differentiation in Chinese tongue sole (*Cynoglossus semilaevis*). Aquaculture and Fisheries; 2024.

[76] Liu J, Mahe S, Guiguen Y. Expression of the T-box transcription factor, T-box 1 (*tbx1*), during rainbow trout (*Oncorhynchus mykiss*) testicular differentiation. Cybium. 2008;32:97.

[77] Yano A, Nicol B, Guerin A, Guiguen Y. The duplicated rainbow trout (Oncorhynchus mykiss) T-box transcription factors 1, tbx1a and tbx1b, are up‐regulated during testicular development. Mol Reprod Dev. 2011;78:172–80. 21308851 10.1002/mrd.21279

[78] Hu Q, Meng Y, Wang D, Tian H, Xiao H. Characterization and function of the *T-box 1* gene in Chinese giant salamander *Andrias Davidianus*. Genomics. 2019;111:1351–9. 30244141 10.1016/j.ygeno.2018.09.007

[79] Wan G, Zhang H, Wang P, Qin Q, Zhou X, Xiong G, et al. Gonadal transcriptome analysis reveals that *SOX17* and *CYP26A1* are involved in sex differentiation in the Chinese soft-shelled turtle (*Pelodiscus sinensis*). Biochem Genet. 2025;63:2190–210. 38710962 10.1007/s10528-024-10815-4

[80] Yang S, Tang X, Yan F, Yang H, Xu L, Jian Z, et al. A time-course transcriptome analysis revealing the potential molecular mechanism of early gonadal differentiation in the Chinese giant salamander. Comp Biochem Phys D. 2024;50:101200. 10.1016/j.cbd.2024.101200 38320446

[81] Page-McCaw A, Ewald AJ, Werb Z. Matrix metalloproteinases and the regulation of tissue remodelling. Nat Rev Mol Cell Bio. 2007;8:221–33. 17318226 10.1038/nrm2125 PMC2760082

[82] Carver JJ, Zhu Y. Metzincin metalloproteases in PGC migration and gonadal sex conversion. Gen Comp Endocrinol. 2023;330:114137. 36191636 10.1016/j.ygcen.2022.114137

[83] Díez-Torre A, Díaz‐Núñez M, Eguizábal C, Silván U, Aréchaga J. Evidence for a role of matrix metalloproteinases and their inhibitors in primordial germ cell migration. Andrology. 2013;1:779–86. 23843195 10.1111/j.2047-2927.2013.00109.x

[84] Mazzoni TS, Quagio-Grassiotto I. Presence of the matrix metalloproteinases during the migration of the primordial germ cells in zebrafish gonadal ridge. Cell Tissue Res. 2021;383:707–22. 32960354 10.1007/s00441-020-03288-5

[85] Mazzoni TS, Lo Nostro FL, Antoneli FN, Quagio-Grassiotto I. Action of the metalloproteinases in gonadal remodeling during sex reversal in the sequential hermaphroditism of the teleostei fish *Synbranchus marmoratus* (Synbranchiformes: Synbranchidae). Cells. 2018;7:34. 29695033 10.3390/cells7050034 PMC5981258

[86] Adolfi MC, Herpin A, Regensburger M, Sacquegno J, Waxman JS, Schartl M. Retinoic acid and meiosis induction in adult versus embryonic gonads of Medaka. Sci Rep. 2016;6:34281. 27677591 10.1038/srep34281 PMC5039705

[87] Schleif MC, Havel SL, Griswold MD. Function of retinoic acid in development of male and female gametes. Nutrients. 2022;14:1293. 35334951 10.3390/nu14061293 PMC8951023

