# Supplementary material for: Sex-specific gonadal transcriptome during early development of Siberian sturgeon
Source: Biol Sex Differ. 2026 Feb 2;17:17. doi: 10.1186/s13293-025-00810-8 (PMC12866037; doi:10.1186/s13293-025-00810-8)
Supplement: Supplementary file 1 — Supplementary Material 1 [file 13293_2025_810_MOESM1_ESM.docx]

Additional file 1. Characteristics of fish used for transcriptomic experiment

| Sample | Length (cm) | Weight (g) | Sex |
| --- | --- | --- | --- |
| 2139 | 11.7 | 5.38 | Fem |
| 2140 | 13.2 | 7.82 | Fem |
| 2141 | 11.2 | 4.78 | Fem |
| 2142 | 10.7 | 4.86 | Fem |
| 2143 | 11.1 | 5.04 | Fem |
| 2145 | 12.2 | 5.59 | Fem |
| 2148 | 11.3 | 4.99 | Mal |
| 2149 | 11.5 | 5.37 | Mal |
| 2150 | 12.2 | 6.87 | Mal |
| 2151 | 10.3 | 4.15 | Mal |
| 2153 | 10.5 | 6.25 | Mal |
| 2175 | 10.8 | 4.7 | Mal |
