# Supplementary material for: Sex-specific gonadal transcriptome during early development of Siberian sturgeon
Source: Biol Sex Differ. 2026 Feb 2;17:17. doi: 10.1186/s13293-025-00810-8 (PMC12866037; doi:10.1186/s13293-025-00810-8)
Supplement: Supplementary file 2 — Supplementary Material 2 [file 13293_2025_810_MOESM2_ESM.docx]

Additional file 2. RIN values of samples used for transcriptomic experiment

| Sample | RIN MacroGen | Sex |
| --- | --- | --- |
| 2139 | 9.3 | Fem |
| 2140 | 9.3 | Fem |
| 2141 | 9.6 | Fem |
| 2142 | 9.2 | Fem |
| 2143 | 9.4 | Fem |
| 2145 | 9.3 | Fem |
| 2148 | 9.2 | Mal |
| 2149 | 8.7 | Mal |
| 2150 | 8.8 | Mal |
| 2151 | 9.3 | Mal |
| 2153 | 8.8 | Mal |
| 2175 | 8.6 | Mal |
