# Supplementary material for: Sex-specific gonadal transcriptome during early development of Siberian sturgeon
Source: Biol Sex Differ. 2026 Feb 2;17:17. doi: 10.1186/s13293-025-00810-8 (PMC12866037; doi:10.1186/s13293-025-00810-8)
Supplement: Supplementary file 3 — Supplementary Material 3 [file 13293_2025_810_MOESM3_ESM.docx]

Additional file 3. All female enriched GO terms

| GO.ID | Term | Process | Adjusted p-value |
| --- | --- | --- | --- |
| GO:0051412 | response to corticosterone | Biological Process | 2.90E-05 |
| GO:0051385 | response to mineralocorticoid | Biological Process | 1.05E-04 |
| GO:0120255 | olefinic compound biosynthetic process | Biological Process | 6.00E-04 |
| GO:0051591 | response to cAMP | Biological Process | 6.00E-04 |
| GO:0030186 | melatonin metabolic process | Biological Process | 1.43E-03 |
| GO:0061370 | testosterone biosynthetic process | Biological Process | 1.43E-03 |
| GO:0046683 | response to organophosphorus | Biological Process | 1.43E-03 |
| GO:0042446 | hormone biosynthetic process | Biological Process | 1.69E-03 |
| GO:0014074 | response to purine-containing compound | Biological Process | 2.11E-03 |
| GO:0006703 | estrogen biosynthetic process | Biological Process | 2.58E-03 |
| GO:0120178 | steroid hormone biosynthetic process | Biological Process | 2.58E-03 |
| GO:1902075 | cellular response to salt | Biological Process | 2.58E-03 |
| GO:0060136 | embryonic process involved in female pregnancy | Biological Process | 2.58E-03 |
| GO:0009636 | response to toxic substance | Biological Process | 3.75E-03 |
| GO:0042181 | ketone biosynthetic process | Biological Process | 3.75E-03 |
| GO:0009314 | response to radiation | Biological Process | 3.75E-03 |
| GO:0051253 | negative regulation of RNA metabolic pro... | Biological Process | 4.12E-03 |
| GO:0032570 | response to progesterone | Biological Process | 6.32E-03 |
| GO:0010038 | response to metal ion | Biological Process | 6.32E-03 |
| GO:0051592 | response to calcium ion | Biological Process | 6.67E-03 |
| GO:1902074 | response to salt | Biological Process | 6.67E-03 |
| GO:1901654 | response to ketone | Biological Process | 6.67E-03 |
| GO:0045934 | negative regulation of nucleobase-contai... | Biological Process | 6.67E-03 |
| GO:1902679 | negative regulation of RNA biosynthetic ... | Biological Process | 6.67E-03 |
| GO:0120254 | olefinic compound metabolic process | Biological Process | 7.50E-03 |
| GO:0045814 | negative regulation of gene expression, ... | Biological Process | 7.50E-03 |
| GO:0051384 | response to glucocorticoid | Biological Process | 8.50E-03 |
| GO:0030316 | osteoclast differentiation | Biological Process | 8.50E-03 |
| GO:0042445 | hormone metabolic process | Biological Process | 8.50E-03 |
| GO:0010035 | response to inorganic substance | Biological Process | 8.50E-03 |
| GO:0071372 | cellular response to follicle-stimulatin... | Biological Process | 8.75E-03 |
| GO:0071277 | cellular response to calcium ion | Biological Process | 8.75E-03 |
| GO:0008210 | estrogen metabolic process | Biological Process | 8.75E-03 |
| GO:0031960 | response to corticosteroid | Biological Process | 8.75E-03 |
| GO:0001829 | trophectodermal cell differentiation | Biological Process | 8.75E-03 |
| GO:0030237 | female sex determination | Biological Process | 8.75E-03 |
| GO:0071248 | cellular response to metal ion | Biological Process | 9.19E-03 |
| GO:0097305 | response to alcohol | Biological Process | 9.21E-03 |
| GO:0060716 | labyrinthine layer blood vessel developm... | Biological Process | 1.08E-02 |
| GO:0032354 | response to follicle-stimulating hormone | Biological Process | 1.10E-02 |
| GO:0009416 | response to light stimulus | Biological Process | 1.26E-02 |
| GO:0071241 | cellular response to inorganic substance | Biological Process | 1.26E-02 |
| GO:0006710 | androgen catabolic process | Biological Process | 1.26E-02 |
| GO:0010164 | response to cesium ion | Biological Process | 1.26E-02 |
| GO:0072303 | positive regulation of glomerular metane... | Biological Process | 1.26E-02 |
| GO:0048545 | response to steroid hormone | Biological Process | 1.42E-02 |
| GO:0045892 | negative regulation of DNA-templated tra... | Biological Process | 1.42E-02 |
| GO:0042430 | indole-containing compound metabolic pro... | Biological Process | 1.42E-02 |
| GO:0046697 | decidualization | Biological Process | 1.42E-02 |
| GO:0002573 | myeloid leukocyte differentiation | Biological Process | 1.48E-02 |
| GO:0071371 | cellular response to gonadotropin stimul... | Biological Process | 1.48E-02 |
| GO:0033687 | osteoblast proliferation | Biological Process | 1.57E-02 |
| GO:0008207 | C21-steroid hormone metabolic process | Biological Process | 1.59E-02 |
| GO:0002686 | negative regulation of leukocyte migrati... | Biological Process | 1.74E-02 |
| GO:0060674 | placenta blood vessel development | Biological Process | 1.85E-02 |
| GO:0001825 | blastocyst formation | Biological Process | 1.85E-02 |
| GO:0010817 | regulation of hormone levels | Biological Process | 1.85E-02 |
| GO:0036146 | cellular response to mycotoxin | Biological Process | 1.85E-02 |
| GO:0060014 | granulosa cell differentiation | Biological Process | 1.85E-02 |
| GO:0071505 | response to mycophenolic acid | Biological Process | 1.85E-02 |
| GO:0071506 | cellular response to mycophenolic acid | Biological Process | 1.85E-02 |
| GO:0032611 | interleukin-1 beta production | Biological Process | 1.89E-02 |
| GO:0001893 | maternal placenta development | Biological Process | 1.91E-02 |
| GO:0009612 | response to mechanical stimulus | Biological Process | 1.91E-02 |
| GO:0060065 | uterus development | Biological Process | 1.93E-02 |
| GO:0032612 | interleukin-1 production | Biological Process | 2.09E-02 |
| GO:0033120 | positive regulation of RNA splicing | Biological Process | 2.32E-02 |
| GO:0002074 | extraocular skeletal muscle development | Biological Process | 2.32E-02 |
| GO:0060280 | negative regulation of ovulation | Biological Process | 2.32E-02 |
| GO:0071492 | cellular response to UV-A | Biological Process | 2.32E-02 |
| GO:0072301 | regulation of metanephric glomerular mes... | Biological Process | 2.32E-02 |
| GO:0098758 | response to interleukin-8 | Biological Process | 2.32E-02 |
| GO:0098759 | cellular response to interleukin-8 | Biological Process | 2.32E-02 |
| GO:0006694 | steroid biosynthetic process | Biological Process | 2.49E-02 |
| GO:0044060 | regulation of endocrine process | Biological Process | 2.50E-02 |
| GO:0034698 | response to gonadotropin | Biological Process | 2.75E-02 |
| GO:0060986 | endocrine hormone secretion | Biological Process | 2.75E-02 |
| GO:0009439 | cyanate metabolic process | Biological Process | 2.75E-02 |
| GO:0009440 | cyanate catabolic process | Biological Process | 2.75E-02 |
| GO:0033686 | positive regulation of luteinizing hormone | Biological Process | 2.75E-02 |
| GO:2000395 | regulation of ubiquitin-dependent endocy... | Biological Process | 2.75E-02 |
| GO:2000397 | positive regulation of ubiquitin-depende... | Biological Process | 2.75E-02 |
| GO:1901362 | organic cyclic compound biosynthetic pro... | Biological Process | 2.99E-02 |
| GO:0007519 | skeletal muscle tissue development | Biological Process | 2.99E-02 |
| GO:0014072 | response to isoquinoline alkaloid | Biological Process | 2.99E-02 |
| GO:0035914 | skeletal muscle cell differentiation | Biological Process | 2.99E-02 |
| GO:0002521 | leukocyte differentiation | Biological Process | 2.99E-02 |
| GO:0042180 | cellular ketone metabolic process | Biological Process | 2.99E-02 |
| GO:0018963 | phthalate metabolic process | Biological Process | 2.99E-02 |
| GO:0030187 | melatonin biosynthetic process | Biological Process | 2.99E-02 |
| GO:0033684 | regulation of luteinizing hormone secretion | Biological Process | 2.99E-02 |
| GO:0072223 | metanephric glomerular mesangium develop... | Biological Process | 2.99E-02 |
| GO:0072262 | metanephric glomerular mesangial cell pr... | Biological Process | 2.99E-02 |
| GO:2000182 | regulation of progesterone biosynthetic ... | Biological Process | 2.99E-02 |
| GO:0060711 | labyrinthine layer development | Biological Process | 3.27E-02 |
| GO:0019221 | cytokine-mediated signaling pathway | Biological Process | 3.31E-02 |
| GO:0006338 | chromatin remodeling | Biological Process | 3.31E-02 |
| GO:0002677 | negative regulation of chronic inflammat... | Biological Process | 3.31E-02 |
| GO:0018894 | dibenzo-p-dioxin metabolic process | Biological Process | 3.31E-02 |
| GO:0035928 | rRNA import into mitochondrion | Biological Process | 3.31E-02 |
| GO:0046219 | indolalkylamine biosynthetic process | Biological Process | 3.31E-02 |
| GO:0071504 | cellular response to heparin | Biological Process | 3.31E-02 |
| GO:0040029 | epigenetic regulation of gene expression | Biological Process | 3.60E-02 |
| GO:0010760 | negative regulation of macrophage chemot... | Biological Process | 3.60E-02 |
| GO:0032275 | luteinizing hormone secretion | Biological Process | 3.60E-02 |
| GO:0035927 | RNA import into mitochondrion | Biological Process | 3.60E-02 |
| GO:0046881 | positive regulation of follicle-stimulat... | Biological Process | 3.60E-02 |
| GO:0071503 | response to heparin | Biological Process | 3.60E-02 |
| GO:0072126 | positive regulation of glomerular mesang... | Biological Process | 3.60E-02 |
| GO:0060135 | maternal process involved in female preg... | Biological Process | 3.60E-02 |
| GO:0010558 | negative regulation of macromolecule bio... | Biological Process | 3.63E-02 |
| GO:0009092 | homoserine metabolic process | Biological Process | 3.71E-02 |
| GO:0009403 | toxin biosynthetic process | Biological Process | 3.71E-02 |
| GO:0010046 | response to mycotoxin | Biological Process | 3.71E-02 |
| GO:0019346 | transsulfuration | Biological Process | 3.71E-02 |
| GO:0032278 | positive regulation of gonadotropin secr... | Biological Process | 3.71E-02 |
| GO:0042435 | indole-containing compound biosynthetic ... | Biological Process | 3.71E-02 |
| GO:0046880 | regulation of follicle-stimulating hormo... | Biological Process | 3.71E-02 |
| GO:0070814 | hydrogen sulfide biosynthetic process | Biological Process | 3.71E-02 |
| GO:1903237 | negative regulation of leukocyte tetheri... | Biological Process | 3.71E-02 |
| GO:2000866 | positive regulation of estradiol secreti... | Biological Process | 3.71E-02 |
| GO:0007565 | female pregnancy | Biological Process | 3.74E-02 |
| GO:0031327 | negative regulation of cellular biosynth... | Biological Process | 3.74E-02 |
| GO:0032620 | interleukin-17 production | Biological Process | 3.74E-02 |
| GO:0009890 | negative regulation of biosynthetic proc... | Biological Process | 3.74E-02 |
| GO:0007530 | sex determination | Biological Process | 3.74E-02 |
| GO:0019100 | male germ-line sex determination | Biological Process | 3.74E-02 |
| GO:0043328 | protein transport to vacuole involved in... | Biological Process | 3.74E-02 |
| GO:0046884 | follicle-stimulating hormone secretion | Biological Process | 3.74E-02 |
| GO:0051029 | rRNA transport | Biological Process | 3.74E-02 |
| GO:0070141 | response to UV-A | Biological Process | 3.74E-02 |
| GO:1901724 | positive regulation of cell proliferatio... | Biological Process | 3.74E-02 |
| GO:1904995 | negative regulation of leukocyte adhesio... | Biological Process | 3.74E-02 |
| GO:1905522 | negative regulation of macrophage migrat... | Biological Process | 3.74E-02 |
| GO:2000864 | regulation of estradiol secretion | Biological Process | 3.74E-02 |
| GO:0071320 | cellular response to cAMP | Biological Process | 3.76E-02 |
| GO:0050886 | endocrine process | Biological Process | 3.90E-02 |
| GO:0042698 | ovulation cycle | Biological Process | 3.90E-02 |
| GO:0002676 | regulation of chronic inflammatory respo... | Biological Process | 3.90E-02 |
| GO:0006701 | progesterone biosynthetic process | Biological Process | 3.90E-02 |
| GO:0032276 | regulation of gonadotropin secretion | Biological Process | 3.90E-02 |
| GO:0035938 | estradiol secretion | Biological Process | 3.90E-02 |
| GO:0072124 | regulation of glomerular mesangial cell ... | Biological Process | 3.90E-02 |
| GO:0072239 | metanephric glomerulus vasculature devel... | Biological Process | 3.90E-02 |
| GO:0043484 | regulation of RNA splicing | Biological Process | 4.08E-02 |
| GO:0019101 | female somatic sex determination | Biological Process | 4.08E-02 |
| GO:0061476 | response to anticoagulant | Biological Process | 4.08E-02 |
| GO:0071464 | cellular response to hydrostatic pressur... | Biological Process | 4.08E-02 |
| GO:0072110 | glomerular mesangial cell proliferation | Biological Process | 4.08E-02 |
| GO:0072300 | positive regulation of metanephric glome... | Biological Process | 4.08E-02 |
| GO:0008202 | steroid metabolic process | Biological Process | 4.08E-02 |
| GO:0044249 | cellular biosynthetic process | Biological Process | 4.08E-02 |
| GO:0033993 | response to lipid | Biological Process | 4.27E-02 |
| GO:0007630 | jump response | Biological Process | 4.31E-02 |
| GO:0034465 | response to carbon monoxide | Biological Process | 4.31E-02 |
| GO:0070086 | ubiquitin-dependent endocytosis | Biological Process | 4.31E-02 |
| GO:0009058 | biosynthetic process | Biological Process | 4.47E-02 |
| GO:0032870 | cellular response to hormone stimulus | Biological Process | 4.47E-02 |
| GO:0001824 | blastocyst development | Biological Process | 4.47E-02 |
| GO:0018879 | biphenyl metabolic process | Biological Process | 4.47E-02 |
| GO:0032274 | gonadotropin secretion | Biological Process | 4.47E-02 |
| GO:0072203 | cell proliferation involved in metanephr... | Biological Process | 4.47E-02 |
| GO:0072298 | regulation of metanephric glomerulus dev... | Biological Process | 4.47E-02 |
| GO:0009617 | response to bacterium | Biological Process | 4.56E-02 |
| GO:0051252 | regulation of RNA metabolic process | Biological Process | 4.56E-02 |
| GO:0034097 | response to cytokine | Biological Process | 4.56E-02 |
| GO:0001661 | conditioned taste aversion | Biological Process | 4.56E-02 |
| GO:0032511 | late endosome to vacuole transport via m... | Biological Process | 4.56E-02 |
| GO:0070813 | hydrogen sulfide metabolic process | Biological Process | 4.56E-02 |
| GO:0071462 | cellular response to water stimulus | Biological Process | 4.56E-02 |
| GO:0071873 | response to norepinephrine | Biological Process | 4.56E-02 |
| GO:1901722 | regulation of cell proliferation involve... | Biological Process | 4.56E-02 |
| GO:0043434 | response to peptide hormone | Biological Process | 4.60E-02 |
| GO:0051172 | negative regulation of nitrogen compound... | Biological Process | 4.72E-02 |
| GO:0072109 | glomerular mesangium development | Biological Process | 4.72E-02 |
| GO:1900194 | negative regulation of oocyte maturation | Biological Process | 4.72E-02 |
| GO:1902949 | positive regulation of tau-protein kinas... | Biological Process | 4.72E-02 |
| GO:0006355 | regulation of DNA-templated transcriptio... | Biological Process | 4.72E-02 |
| GO:0001570 | vasculogenesis | Biological Process | 4.72E-02 |
| GO:0044706 | multi-multicellular organism process | Biological Process | 4.91E-02 |
| GO:0002692 | negative regulation of cellular extravas... | Biological Process | 4.95E-02 |
| GO:0030099 | myeloid cell differentiation | Biological Process | 4.95E-02 |
| GO:0008380 | RNA splicing | Biological Process | 4.95E-02 |
| GO:0010243 | response to organonitrogen compound | Biological Process | 4.95E-02 |
| GO:0060538 | skeletal muscle organ development | Biological Process | 4.99E-02 |
| GO:0014070 | response to organic cyclic compound | Biological Process | 4.99E-02 |
| GO:0035976 | transcription factor AP-1 complex | Cellular Component | 1.25E-05 |
| GO:0000228 | nuclear chromosome | Cellular Component | 5.25E-03 |
| GO:0032993 | protein-DNA complex | Cellular Component | 2.00E-02 |
| GO:1901363 | heterocyclic compound binding | Molecular Function | 7.25E-03 |
| GO:0097159 | organic cyclic compound binding | Molecular Function | 7.25E-03 |
| GO:0003676 | nucleic acid binding | Molecular Function | 1.17E-02 |
| GO:0001216 | DNA-binding transcription activator activity | Molecular Function | 1.17E-02 |
| GO:0001228 | DNA-binding transcription activator activity RNA polymerase II-specific | Molecular Function | 1.17E-02 |
| GO:0003677 | DNA binding | Molecular Function | 1.17E-02 |
