# Supplementary material for: Sex-specific gonadal transcriptome during early development of Siberian sturgeon
Source: Biol Sex Differ. 2026 Feb 2;17:17. doi: 10.1186/s13293-025-00810-8 (PMC12866037; doi:10.1186/s13293-025-00810-8)
Supplement: Supplementary file 4 — Supplementary Material 4 [file 13293_2025_810_MOESM4_ESM.docx]

Additional file 4. All male enriched GO terms

| GO.ID | Term | Process | Adjusted p-value |
| --- | --- | --- | --- |
| GO:0044258 | intestinal lipid catabolic process | Biological Process | 2,40E-07 |
| GO:0030299 | intestinal cholesterol absorption | Biological Process | 1,95E-05 |
| GO:0015850 | organic hydroxy compound transport | Biological Process | 2,63E-05 |
| GO:0044241 | lipid digestion | Biological Process | 2,63E-05 |
| GO:0098856 | intestinal lipid absorption | Biological Process | 5,40E-05 |
| GO:0006869 | lipid transport | Biological Process | 2,08E-04 |
| GO:0046466 | membrane lipid catabolic process | Biological Process | 2,36E-04 |
| GO:0006706 | steroid catabolic process | Biological Process | 2,69E-04 |
| GO:0050892 | intestinal absorption | Biological Process | 6,67E-04 |
| GO:0010876 | lipid localization | Biological Process | 7,50E-04 |
| GO:0071938 | vitamin A transport | Biological Process | 1,37E-03 |
| GO:0016042 | lipid catabolic process | Biological Process | 1,37E-03 |
| GO:0001523 | retinoid metabolic process | Biological Process | 1,37E-03 |
| GO:0007586 | digestion | Biological Process | 1,37E-03 |
| GO:0034633 | retinol transport | Biological Process | 1,37E-03 |
| GO:0046864 | isoprenoid transport | Biological Process | 1,37E-03 |
| GO:0046865 | terpenoid transport | Biological Process | 1,37E-03 |
| GO:0097118 | neuroligin clustering involved in postsy... | Biological Process | 1,37E-03 |
| GO:0016101 | diterpenoid metabolic process | Biological Process | 1,37E-03 |
| GO:0010041 | response to iron(III) ion | Biological Process | 1,68E-03 |
| GO:0006721 | terpenoid metabolic process | Biological Process | 2,00E-03 |
| GO:0035176 | social behavior | Biological Process | 2,27E-03 |
| GO:0051703 | biological process involved in intraspec... | Biological Process | 2,39E-03 |
| GO:0030301 | cholesterol transport | Biological Process | 2,59E-03 |
| GO:0042572 | retinol metabolic process | Biological Process | 2,59E-03 |
| GO:0120254 | olefinic compound metabolic process | Biological Process | 2,59E-03 |
| GO:0006707 | cholesterol catabolic process | Biological Process | 2,59E-03 |
| GO:0016127 | sterol catabolic process | Biological Process | 2,59E-03 |
| GO:0046514 | ceramide catabolic process | Biological Process | 2,59E-03 |
| GO:0015918 | sterol transport | Biological Process | 2,67E-03 |
| GO:0097104 | postsynaptic membrane assembly | Biological Process | 3,39E-03 |
| GO:0007567 | parturition | Biological Process | 3,82E-03 |
| GO:0030149 | sphingolipid catabolic process | Biological Process | 3,82E-03 |
| GO:0006720 | isoprenoid metabolic process | Biological Process | 3,82E-03 |
| GO:0071625 | vocalization behavior | Biological Process | 4,00E-03 |
| GO:0097105 | presynaptic membrane assembly | Biological Process | 4,31E-03 |
| GO:0030574 | collagen catabolic process | Biological Process | 4,34E-03 |
| GO:0099068 | postsynapse assembly | Biological Process | 4,34E-03 |
| GO:0007158 | neuron cell-cell adhesion | Biological Process | 5,25E-03 |
| GO:0030157 | pancreatic juice secretion | Biological Process | 5,25E-03 |
| GO:1900244 | positive regulation of synaptic vesicle ... | Biological Process | 5,98E-03 |
| GO:0097090 | presynaptic membrane organization | Biological Process | 6,79E-03 |
| GO:1903423 | positive regulation of synaptic vesicle ... | Biological Process | 7,21E-03 |
| GO:0033189 | response to vitamin A | Biological Process | 7,39E-03 |
| GO:1900242 | regulation of synaptic vesicle endocytos... | Biological Process | 7,83E-03 |
| GO:0022600 | digestive system process | Biological Process | 7,83E-03 |
| GO:0009692 | ethylene metabolic process | Biological Process | 8,37E-03 |
| GO:0019341 | dibenzo-p-dioxin catabolic process | Biological Process | 8,37E-03 |
| GO:0043449 | cellular alkene metabolic process | Biological Process | 8,37E-03 |
| GO:0051180 | vitamin transport | Biological Process | 8,82E-03 |
| GO:0071276 | cellular response to cadmium ion | Biological Process | 8,82E-03 |
| GO:1900073 | regulation of neuromuscular synaptic tra... | Biological Process | 8,94E-03 |
| GO:0046903 | secretion | Biological Process | 9,06E-03 |
| GO:0015718 | monocarboxylic acid transport | Biological Process | 1,04E-02 |
| GO:0046164 | alcohol catabolic process | Biological Process | 1,04E-02 |
| GO:0010039 | response to iron ion | Biological Process | 1,04E-02 |
| GO:1903421 | regulation of synaptic vesicle recycling | Biological Process | 1,04E-02 |
| GO:0055093 | response to hyperoxia | Biological Process | 1,24E-02 |
| GO:2000331 | regulation of terminal button organizati... | Biological Process | 1,24E-02 |
| GO:0032094 | response to food | Biological Process | 1,25E-02 |
| GO:0006038 | cell wall chitin biosynthetic process | Biological Process | 1,25E-02 |
| GO:0009624 | response to nematode | Biological Process | 1,25E-02 |
| GO:0019376 | galactolipid catabolic process | Biological Process | 1,25E-02 |
| GO:0043385 | mycotoxin metabolic process | Biological Process | 1,25E-02 |
| GO:0070592 | cell wall polysaccharide biosynthetic pr... | Biological Process | 1,25E-02 |
| GO:0071460 | cellular response to cell-matrix adhesio... | Biological Process | 1,25E-02 |
| GO:0006643 | membrane lipid metabolic process | Biological Process | 1,31E-02 |
| GO:0032941 | secretion by tissue | Biological Process | 1,35E-02 |
| GO:0099054 | presynapse assembly | Biological Process | 1,35E-02 |
| GO:1901616 | organic hydroxy compound catabolic proce... | Biological Process | 1,36E-02 |
| GO:0034308 | primary alcohol metabolic process | Biological Process | 1,36E-02 |
| GO:0036296 | response to increased oxygen levels | Biological Process | 1,36E-02 |
| GO:0030225 | macrophage differentiation | Biological Process | 1,41E-02 |
| GO:0022617 | extracellular matrix disassembly | Biological Process | 1,49E-02 |
| GO:0044242 | cellular lipid catabolic process | Biological Process | 1,51E-02 |
| GO:0042445 | hormone metabolic process | Biological Process | 1,51E-02 |
| GO:0006037 | cell wall chitin metabolic process | Biological Process | 1,51E-02 |
| GO:0021564 | vagus nerve development | Biological Process | 1,51E-02 |
| GO:0021644 | vagus nerve morphogenesis | Biological Process | 1,51E-02 |
| GO:0042546 | cell wall biogenesis | Biological Process | 1,51E-02 |
| GO:0044038 | cell wall macromolecule biosynthetic pro... | Biological Process | 1,51E-02 |
| GO:1900673 | olefin metabolic process | Biological Process | 1,51E-02 |
| GO:0001941 | postsynaptic membrane organization | Biological Process | 1,55E-02 |
| GO:0009056 | catabolic process | Biological Process | 1,62E-02 |
| GO:0031638 | zymogen activation | Biological Process | 1,68E-02 |
| GO:0033013 | tetrapyrrole metabolic process | Biological Process | 1,70E-02 |
| GO:0065008 | regulation of biological quality | Biological Process | 1,73E-02 |
| GO:0035094 | response to nicotine | Biological Process | 1,73E-02 |
| GO:0071709 | membrane assembly | Biological Process | 1,73E-02 |
| GO:0006034 | cuticle chitin metabolic process | Biological Process | 1,73E-02 |
| GO:0006035 | cuticle chitin biosynthetic process | Biological Process | 1,73E-02 |
| GO:0009644 | response to high light intensity | Biological Process | 1,73E-02 |
| GO:0010383 | cell wall polysaccharide metabolic proce... | Biological Process | 1,73E-02 |
| GO:0060023 | soft palate development | Biological Process | 1,73E-02 |
| GO:0072513 | positive regulation of secondary heart f... | Biological Process | 1,73E-02 |
| GO:0099174 | regulation of presynapse organization | Biological Process | 1,78E-02 |
| GO:0001525 | angiogenesis | Biological Process | 1,92E-02 |
| GO:0006672 | ceramide metabolic process | Biological Process | 2,00E-02 |
| GO:0006031 | chitin biosynthetic process | Biological Process | 2,00E-02 |
| GO:0019374 | galactolipid metabolic process | Biological Process | 2,00E-02 |
| GO:0036345 | platelet maturation | Biological Process | 2,00E-02 |
| GO:0042904 | 9-cis-retinoic acid biosynthetic process | Biological Process | 2,00E-02 |
| GO:0042905 | 9-cis-retinoic acid metabolic process | Biological Process | 2,00E-02 |
| GO:0072553 | terminal button organization | Biological Process | 2,02E-02 |
| GO:0010817 | regulation of hormone levels | Biological Process | 2,06E-02 |
| GO:0098693 | regulation of synaptic vesicle cycle | Biological Process | 2,19E-02 |
| GO:0009698 | phenylpropanoid metabolic process | Biological Process | 2,19E-02 |
| GO:0009804 | coumarin metabolic process | Biological Process | 2,19E-02 |
| GO:0017143 | insecticide metabolic process | Biological Process | 2,19E-02 |
| GO:0018894 | dibenzo-p-dioxin metabolic process | Biological Process | 2,19E-02 |
| GO:0035981 | tongue muscle cell differentiation | Biological Process | 2,19E-02 |
| GO:2001035 | regulation of tongue muscle cell differe... | Biological Process | 2,19E-02 |
| GO:2001037 | positive regulation of tongue muscle cel... | Biological Process | 2,19E-02 |
| GO:0043113 | receptor clustering | Biological Process | 2,19E-02 |
| GO:0008202 | steroid metabolic process | Biological Process | 2,20E-02 |
| GO:0044091 | membrane biogenesis | Biological Process | 2,28E-02 |
| GO:0009812 | flavonoid metabolic process | Biological Process | 2,35E-02 |
| GO:0019087 | transformation of host cell by virus | Biological Process | 2,35E-02 |
| GO:0044036 | cell wall macromolecule metabolic proces... | Biological Process | 2,35E-02 |
| GO:0071554 | cell wall organization or biogenesis | Biological Process | 2,35E-02 |
| GO:1901073 | glucosamine-containing compound biosynth... | Biological Process | 2,35E-02 |
| GO:2001268 | negative regulation of cysteine-type end... | Biological Process | 2,35E-02 |
| GO:0046942 | carboxylic acid transport | Biological Process | 2,35E-02 |
| GO:0015849 | organic acid transport | Biological Process | 2,35E-02 |
| GO:0046686 | response to cadmium ion | Biological Process | 2,38E-02 |
| GO:0009403 | toxin biosynthetic process | Biological Process | 2,55E-02 |
| GO:0009820 | alkaloid metabolic process | Biological Process | 2,55E-02 |
| GO:0016098 | monoterpenoid metabolic process | Biological Process | 2,55E-02 |
| GO:1902811 | positive regulation of skeletal muscle f... | Biological Process | 2,55E-02 |
| GO:0060017 | parathyroid gland development | Biological Process | 2,80E-02 |
| GO:0061365 | positive regulation of triglyceride lipa... | Biological Process | 2,80E-02 |
| GO:1902809 | regulation of skeletal muscle fiber diff... | Biological Process | 2,80E-02 |
| GO:0006631 | fatty acid metabolic process | Biological Process | 2,86E-02 |
| GO:0032963 | collagen metabolic process | Biological Process | 2,88E-02 |
| GO:0048514 | blood vessel morphogenesis | Biological Process | 2,88E-02 |
| GO:0002933 | lipid hydroxylation | Biological Process | 3,00E-02 |
| GO:0071283 | cellular response to iron(III) ion | Biological Process | 3,00E-02 |
| GO:0007584 | response to nutrient | Biological Process | 3,00E-02 |
| GO:0006810 | transport | Biological Process | 3,01E-02 |
| GO:0007589 | body fluid secretion | Biological Process | 3,04E-02 |
| GO:0003008 | system process | Biological Process | 3,07E-02 |
| GO:0006811 | monoatomic ion transport | Biological Process | 3,07E-02 |
| GO:0002138 | retinoic acid biosynthetic process | Biological Process | 3,07E-02 |
| GO:0006030 | chitin metabolic process | Biological Process | 3,07E-02 |
| GO:0035745 | T-helper 2 cell cytokine production | Biological Process | 3,07E-02 |
| GO:0042473 | outer ear morphogenesis | Biological Process | 3,07E-02 |
| GO:0098528 | skeletal muscle fiber differentiation | Biological Process | 3,07E-02 |
| GO:0002274 | myeloid leukocyte activation | Biological Process | 3,09E-02 |
| GO:0099172 | presynapse organization | Biological Process | 3,09E-02 |
| GO:0007274 | neuromuscular synaptic transmission | Biological Process | 3,14E-02 |
| GO:0009062 | fatty acid catabolic process | Biological Process | 3,15E-02 |
| GO:0008203 | cholesterol metabolic process | Biological Process | 3,16E-02 |
| GO:0051549 | positive regulation of keratinocyte migr... | Biological Process | 3,16E-02 |
| GO:0060137 | maternal process involved in parturition | Biological Process | 3,16E-02 |
| GO:0097267 | omega-hydroxylase P450 pathway | Biological Process | 3,16E-02 |
| GO:2001016 | positive regulation of skeletal muscle c... | Biological Process | 3,16E-02 |
| GO:0006766 | vitamin metabolic process | Biological Process | 3,22E-02 |
| GO:0019373 | epoxygenase P450 pathway | Biological Process | 3,38E-02 |
| GO:0003266 | regulation of secondary heart field card... | Biological Process | 3,53E-02 |
| GO:0043031 | negative regulation of macrophage activa... | Biological Process | 3,53E-02 |
| GO:0043282 | pharyngeal muscle development | Biological Process | 3,53E-02 |
| GO:0060982 | coronary artery morphogenesis | Biological Process | 3,53E-02 |
| GO:0071347 | cellular response to interleukin-1 | Biological Process | 3,53E-02 |
| GO:0072329 | monocarboxylic acid catabolic process | Biological Process | 3,74E-02 |
| GO:0051234 | establishment of localization | Biological Process | 3,74E-02 |
| GO:0033273 | response to vitamin | Biological Process | 3,74E-02 |
| GO:0048488 | synaptic vesicle endocytosis | Biological Process | 3,74E-02 |
| GO:0140238 | presynaptic endocytosis | Biological Process | 3,74E-02 |
| GO:0048565 | digestive tract development | Biological Process | 3,74E-02 |
| GO:0006665 | sphingolipid metabolic process | Biological Process | 3,74E-02 |
| GO:0044282 | small molecule catabolic process | Biological Process | 3,74E-02 |
| GO:0001568 | blood vessel development | Biological Process | 3,74E-02 |
| GO:0033993 | response to lipid | Biological Process | 3,74E-02 |
| GO:0042178 | xenobiotic catabolic process | Biological Process | 3,74E-02 |
| GO:0070166 | enamel mineralization | Biological Process | 3,74E-02 |
| GO:0051179 | localization | Biological Process | 3,74E-02 |
| GO:1902652 | secondary alcohol metabolic process | Biological Process | 3,77E-02 |
| GO:0030198 | extracellular matrix organization | Biological Process | 3,83E-02 |
| GO:0042221 | response to chemical | Biological Process | 3,83E-02 |
| GO:0060627 | regulation of vesicle-mediated transport | Biological Process | 3,83E-02 |
| GO:0071280 | cellular response to copper ion | Biological Process | 3,83E-02 |
| GO:0090331 | negative regulation of platelet aggregat... | Biological Process | 3,83E-02 |
| GO:1901575 | organic substance catabolic process | Biological Process | 3,90E-02 |
| GO:0016125 | sterol metabolic process | Biological Process | 3,90E-02 |
| GO:0016102 | diterpenoid biosynthetic process | Biological Process | 3,90E-02 |
| GO:0017000 | antibiotic biosynthetic process | Biological Process | 3,90E-02 |
| GO:0046349 | amino sugar biosynthetic process | Biological Process | 3,90E-02 |
| GO:1900122 | positive regulation of receptor binding | Biological Process | 3,90E-02 |
| GO:0032940 | secretion by cell | Biological Process | 3,90E-02 |
| GO:0055123 | digestive system development | Biological Process | 3,90E-02 |
| GO:0035296 | regulation of tube diameter | Biological Process | 3,90E-02 |
| GO:0036465 | synaptic vesicle recycling | Biological Process | 3,90E-02 |
| GO:0003263 | cardioblast proliferation | Biological Process | 3,90E-02 |
| GO:0003264 | regulation of cardioblast proliferation | Biological Process | 3,90E-02 |
| GO:0009642 | response to light intensity | Biological Process | 3,90E-02 |
| GO:0051547 | regulation of keratinocyte migration | Biological Process | 3,90E-02 |
| GO:0060586 | multicellular organismal-level iron ion ... | Biological Process | 3,90E-02 |
| GO:0098719 | sodium ion import across plasma membrane | Biological Process | 3,90E-02 |
| GO:0031667 | response to nutrient levels | Biological Process | 3,90E-02 |
| GO:0015711 | organic anion transport | Biological Process | 3,90E-02 |
| GO:0001944 | vasculature development | Biological Process | 3,90E-02 |
| GO:0032526 | response to retinoic acid | Biological Process | 3,90E-02 |
| GO:0016192 | vesicle-mediated transport | Biological Process | 3,90E-02 |
| GO:0140352 | export from cell | Biological Process | 3,93E-02 |
| GO:0070365 | hepatocyte differentiation | Biological Process | 3,99E-02 |
| GO:0050807 | regulation of synapse organization | Biological Process | 3,99E-02 |
| GO:0031915 | positive regulation of synaptic plastici... | Biological Process | 4,15E-02 |
| GO:0009991 | response to extracellular stimulus | Biological Process | 4,17E-02 |
| GO:0002443 | leukocyte mediated immunity | Biological Process | 4,17E-02 |
| GO:0014070 | response to organic cyclic compound | Biological Process | 4,25E-02 |
| GO:2001054 | negative regulation of mesenchymal cell ... | Biological Process | 4,25E-02 |
| GO:0099173 | postsynapse organization | Biological Process | 4,25E-02 |
| GO:0070555 | response to interleukin-1 | Biological Process | 4,29E-02 |
| GO:0007416 | synapse assembly | Biological Process | 4,29E-02 |
| GO:0019377 | glycolipid catabolic process | Biological Process | 4,29E-02 |
| GO:0034116 | positive regulation of heterotypic cell-... | Biological Process | 4,29E-02 |
| GO:0035743 | CD4-positive, alpha-beta T cell cytokine... | Biological Process | 4,29E-02 |
| GO:0042474 | middle ear morphogenesis | Biological Process | 4,29E-02 |
| GO:0042573 | retinoic acid metabolic process | Biological Process | 4,29E-02 |
| GO:0045055 | regulated exocytosis | Biological Process | 4,30E-02 |
| GO:0050878 | regulation of body fluid levels | Biological Process | 4,32E-02 |
| GO:0016114 | terpenoid biosynthetic process | Biological Process | 4,32E-02 |
| GO:0050665 | hydrogen peroxide biosynthetic process | Biological Process | 4,32E-02 |
| GO:0060536 | cartilage morphogenesis | Biological Process | 4,32E-02 |
| GO:0071281 | cellular response to iron ion | Biological Process | 4,32E-02 |
| GO:0097186 | amelogenesis | Biological Process | 4,32E-02 |
| GO:2001053 | regulation of mesenchymal cell apoptotic... | Biological Process | 4,32E-02 |
| GO:0043062 | extracellular structure organization | Biological Process | 4,34E-02 |
| GO:0050803 | regulation of synapse structure or activ... | Biological Process | 4,34E-02 |
| GO:0034111 | negative regulation of homotypic cell-ce... | Biological Process | 4,42E-02 |
| GO:0090330 | regulation of platelet aggregation | Biological Process | 4,42E-02 |
| GO:0009605 | response to external stimulus | Biological Process | 4,49E-02 |
| GO:0043587 | tongue morphogenesis | Biological Process | 4,52E-02 |
| GO:0048752 | semicircular canal morphogenesis | Biological Process | 4,52E-02 |
| GO:0120252 | hydrocarbon metabolic process | Biological Process | 4,52E-02 |
| GO:0050804 | modulation of chemical synaptic transmis... | Biological Process | 4,62E-02 |
| GO:0099177 | regulation of trans-synaptic signaling | Biological Process | 4,62E-02 |
| GO:0097152 | mesenchymal cell apoptotic process | Biological Process | 4,62E-02 |
| GO:0008306 | associative learning | Biological Process | 4,62E-02 |
| GO:0035725 | sodium ion transmembrane transport | Biological Process | 4,66E-02 |
| GO:0031639 | plasminogen activation | Biological Process | 4,74E-02 |
| GO:0071702 | organic substance transport | Biological Process | 4,80E-02 |
| GO:0043312 | neutrophil degranulation | Biological Process | 4,83E-02 |
| GO:0045229 | external encapsulating structure organiz... | Biological Process | 4,83E-02 |
| GO:0035158 | regulation of tube diameter, open trache... | Biological Process | 4,83E-02 |
| GO:0002283 | neutrophil activation involved in immune... | Biological Process | 4,84E-02 |
| GO:0010544 | negative regulation of platelet activati... | Biological Process | 4,93E-02 |
| GO:0070989 | oxidative demethylation | Biological Process | 4,93E-02 |
| GO:0098655 | monoatomic cation transmembrane transpor... | Biological Process | 4,93E-02 |
| GO:0042119 | neutrophil activation | Biological Process | 4,95E-02 |
| GO:0009235 | cobalamin metabolic process | Biological Process | 4,95E-02 |
| GO:0009404 | toxin metabolic process | Biological Process | 4,95E-02 |
| GO:0034505 | tooth mineralization | Biological Process | 4,95E-02 |
| GO:0051546 | keratinocyte migration | Biological Process | 4,95E-02 |
| GO:0071600 | otic vesicle morphogenesis | Biological Process | 4,95E-02 |
| GO:0002446 | neutrophil mediated immunity | Biological Process | 4,99E-02 |
| GO:0005615 | extracellular space | Cellular Component | 1,30E-05 |
| GO:0005576 | extracellular region | Cellular Component | 2,75E-05 |
| GO:0042588 | zymogen granule | Cellular Component | 3,17E-04 |
| GO:0060102 | collagen and cuticulin-based cuticle ext... | Cellular Component | 4,75E-04 |
| GO:0098820 | trans-synaptic protein complex | Cellular Component | 1,30E-03 |
| GO:0030141 | secretory granule | Cellular Component | 3,17E-03 |
| GO:0099503 | secretory vesicle | Cellular Component | 7,86E-03 |
| GO:0047372 | acylglycerol lipase activity | Molecular Function | 4,17E-04 |
| GO:0004806 | triglyceride lipase activity | Molecular Function | 4,17E-04 |
| GO:0050253 | retinyl-palmitate esterase activity | Molecular Function | 4,17E-04 |
| GO:0008237 | metallopeptidase activity | Molecular Function | 3,70E-03 |
| GO:0097109 | neuroligin family protein binding | Molecular Function | 3,70E-03 |
| GO:0004771 | sterol esterase activity | Molecular Function | 3,83E-03 |
| GO:0043208 | glycosphingolipid binding | Molecular Function | 1,50E-02 |
| GO:0004620 | phospholipase activity | Molecular Function | 1,71E-02 |
| GO:0004181 | metallocarboxypeptidase activity | Molecular Function | 1,71E-02 |
| GO:0004622 | lysophospholipase activity | Molecular Function | 1,71E-02 |
| GO:0052689 | carboxylic ester hydrolase activity | Molecular Function | 1,71E-02 |
| GO:0034185 | apolipoprotein binding | Molecular Function | 1,71E-02 |
| GO:0051861 | glycolipid binding | Molecular Function | 1,71E-02 |
| GO:0016298 | lipase activity | Molecular Function | 1,71E-02 |
| GO:0046625 | sphingolipid binding | Molecular Function | 1,90E-02 |
| GO:0004180 | carboxypeptidase activity | Molecular Function | 2,00E-02 |
| GO:0016711 | flavonoid 3'-monooxygenase activity | Molecular Function | 2,53E-02 |
| GO:0047714 | galactolipase activity | Molecular Function | 2,53E-02 |
| GO:0042043 | neurexin family protein binding | Molecular Function | 2,53E-02 |
| GO:0005246 | calcium channel regulator activity | Molecular Function | 2,90E-02 |
| GO:0005539 | glycosaminoglycan binding | Molecular Function | 3,98E-02 |
| GO:0004100 | chitin synthase activity | Molecular Function | 4,27E-02 |
| GO:0008235 | metalloexopeptidase activity | Molecular Function | 4,83E-02 |
