# Supplementary material for: Sex-specific gonadal transcriptome during early development of Siberian sturgeon
Source: Biol Sex Differ. 2026 Feb 2;17:17. doi: 10.1186/s13293-025-00810-8 (PMC12866037; doi:10.1186/s13293-025-00810-8)
Supplement: Supplementary file 5 — Supplementary Material 5 [file 13293_2025_810_MOESM5_ESM.docx]

Additional file 5. Female differentially expressed contigs

| NCBI reference | Log2FC | logCPM | FDR | Id Blastn | ORF / Id Blastp |
| --- | --- | --- | --- | --- | --- |
| GICD01088227.1 | 3,110912 | 0,695855 | 2,18E-11 | hsd17b1 | SI / hsd17b1 |
| GICD01082292.1 | 2,181414 | 0,300276 | 9,16E-09 | neuronal acetylcholine receptor subunit alpha-7 like | SI / neuronal acetylcholine receptor subunit alpha-7 like |
| GICD01027714.1 | 1,514963 | 3,580543 | 4,24E-07 | immediate early response gene 2 protein-like | SI / immediate early response gene 2 protein-like |
| GICD01057103.1 | 2,529127 | 2,386693 | 4,52E-07 | GTP-binding protein Di-Ras2 | NO ORF |
| GICD01032226.1 | 1,52823 | 2,426129 | 7,54E-07 | thyrotropin-releasing hormone receptor-like | NO ORF |
| GICD01064951.1 | 2,405916 | 3,375973 | 8,92E-07 | GTP-binding protein Di-Ras2 | SI / GTP-binding protein Di-Ras2 |
| GICD01077530.1 | 3,013134 | 3,372265 | 8,92E-07 | Protein c-Fos like | SI / proto-oncogene c-Fos-like |
| GICD01090265.1 | 1,958954 | 1,182458 | 9,17E-07 | aromatase (Cyp19a1) | SI / Aromatase (Cyp19a1) |
| GICD01061659.1 | 1,520626 | 3,515408 | 1,63E-06 | *Acipenser ruthenus* genome assembly, chromosome: 36/34 | NO ORF |
| GICD01087373.1 | 1,765854 | 2,896552 | 2,1E-06 | thyrotropin-releasing hormone receptor-like | SI / Thyrotropin-releasing hormone receptor |
| GICD01064885.1 | 1,271667 | 3,496247 | 2,94E-06 | thyrotropin-releasing hormone receptor-like | NO ORF |
| GICD01034201.1 | 2,238274 | 1,725609 | 4,64E-06 | chromosome 36/34 | NO ORF |
| GICD01023208.1 | 2,635137 | 0,455554 | 1,57E-05 | forkhead box protein L2, (Foxl2) | SI / forkhead box protein L2 (Foxl2) |
| GICD01061815.1 | 6,029373 | -2,85895 | 2,58E-05 | Arf GAP with NH3 domaine | NO ORF |
| GICD01029690.1 | 2,996036 | -0,45028 | 8,43E-05 | GTP-binding protein Di-Ras2 | NO ORF |
| GICD01059572.1 | 1,821218 | 3,827602 | 0,000213 | transcription factor JunB-like | SI / transcription factor JunB isoform X1 |
| GICD01049505.1 | 1,315427 | 1,530668 | 0,002342 | immediate early response gene 2 protein-like | NO ORF |
| GICD01085353.1 | 1,321899 | 1,350901 | 0,002834 | ras-like protein family member 10B | NO ORF |
| GICD01077643.1 | 3,186047 | -2,55241 | 0,002834 | *Acipenser ruthenus* genome assembly chromosome 38/41 | NO ORF |
| GICD01062680.1 | 1,398862 | 1,976389 | 0,003001 | reactive oxygen species modulator 1 | NO ORF |
| GICD01016203.1 | 1,607653 | 0,214576 | 0,003562 | uncharacterized ncRNA | NO ORF |
| GICD01005274.1 | 1,546012 | 1,461984 | 0,003565 | transcription factor JunB-like | NO ORF |
| GICD01087320.1 | 1,38849 | 0,997371 | 0,006129 | transmembrane protein 271-like mRNA | SI / transmembrane protein 271-like mRNA |
| GICD01001737.1 | 1,256501 | 0,022116 | 0,006423 | F-box/LRR-repeat protein 12-like mRNA | NO ORF |
| GICD01032392.1 | 1,37497 | 1,343623 | 0,006423 | tonsoku-like protein, mRNA | SI / Tonsoku-like protein |
| GICD01084445.1 | 1,144446 | 5,036742 | 0,007341 | early growth response protein 1-like | SI / early growth response protein 1-like |
| GICD01059511.1 | 1,449407 | 0,104438 | 0,007341 | immediate early response gene 2 protein-like | SI / immediate early response gene 2 protein-like |
| GICD01025436.1 | 1,655932 | 1,328024 | 0,007341 | immediate early response gene 2 protein-like | NO ORF |
| GICD01007426.1 | 4,642036 | 1,464267 | 0,009067 | MHC class 1 alpha antigen | SI / MHC class I alpha antigen |
| GICD01089620.1 | 3,078826 | 1,685651 | 0,009078 | serotonin N-acetyltransferase-like | SI / serotonin N-acetyltransferase-like |
| GICD01053959.1 | 3,411493 | -2,7595 | 0,009078 | Putative lipid scramblase CLPTM1 | NO ORF |
| GICD01019522.1 | 0,921357 | 5,342638 | 0,010673 | uroplakin-2-like transcript variant X2, mRNA | NO ORF |
| GICD01078088.1 | 2,15909 | 0,774713 | 0,014769 | protein c-Fos-like, mRNA | SI /proto-oncogene c-Fos-like isoform X1 |
| GICD01031490.1 | 2,59331 | -1,12732 | 0,018291 | immediate early response gene 2 protein-like | NO ORF |
| GICD01009598.1 | 0,955002 | 5,456288 | 0,01866 | Uncharacterized protein, mRNA | SI / uncharacterized protein LOC131735831 |
| GICD01048872.1 | 1,713444 | 0,366962 | 0,01866 | immediate early response gene 2 protein-like | NO ORF |
| GICD01009655.1 | 8,197664 | 0,035446 | 0,021374 | MHC class lachain (Acsi-UBA) | SI / H-2 class I histocompatibility antigen |
| GICD01009175.1 | 0,90366 | 6,951717 | 0,021791 | uncharacterized LOC117415343 | NO ORF |
| GICD01001726.1 | 1,067252 | 9,102207 | 0,021872 | *Acipenser ruthenus* genome assembly, chromosome: 32 | SI / histone H4-like |
| GICD01066238.1 | 0,857954 | 2,40712 | 0,02521 | tuftelin-like , mRNA | NO ORF |
| GICD01030876.1 | 2,291337 | 0,79341 | 0,025436 | no hay resultado | NO ORF |
| GICD01000328.1 | 1,174873 | 7,749682 | 0,025759 | histone H2A, mRNA | SI / uncharacterized protein LOC121514829 |
| GICD01060537.1 | 6,192481 | -0,58887 | 0,025759 | NHERF family PDZ scaffold protein 1B | NO ORF |
| GICD01086081.1 | 1,354627 | 1,335968 | 0,027046 | neuropeptide Y receptor Y2, like (npy2rl), transcript variant X2 | NO ORF |
| GICD01066315.1 | 0,929594 | 7,595763 | 0,030163 | *Acipenser ruthenus* genome assembly, chromosome: 55 | SI / histone H4-like |
| GICD01015861.1 | 1,330658 | 0,47322 | 0,032135 | L-asparaginase-like, mRNA | SI / L-asparaginase-like |
| GICD01010688.1 | 1,528112 | 2,56403 | 0,033206 | *Acipenser ruthenus* genome assembly, chromosome: 51 | SI / hypothetical protein EOD39_19946 |
| GICD01015009.1 | 0,888127 | 2,095964 | 0,03487 | tubulin alpha chain-like, mRNA | NO ORF |
| GICD01073114.1 | 1,35344 | 4,12034 | 0,038297 | histone H2A, mRNA | SI / H2A protein |
| GICD01030746.1 | 0,808082 | 8,296956 | 0,04469 | *Acipenser ruthenus* genome assembly, chromosome: 20 | SI / histone H2A-like |
| GICD01066742.1 | 0,851523 | 2,603493 | 0,046754 | mercaptopyruvate sulfurtransferase (mpst), mRNA | SI / 3-mercaptopyruvate sulfurtransferase |
| GICD01014721.1 | 0,853944 | 2,971729 | 0,046754 | class I histocompatibility antigen, F10 alpha chain-like | NO ORF |
| GICD01059541.1 | 0,92957 | 2,729705 | 0,046754 | RING finger protein 227-like, mRNA | SI / RING finger protein 227 |
| GICD01014699.1 | 1,029398 | 1,469576 | 0,046754 | cAMP-responsive element modulator-like | NO ORF |
| GICD01059358.1 | 1,032483 | 4,095927 | 0,046754 | *Acipenser ruthenus* genome assembly, chromosome: 14 | NO ORF |
| GICD01080907.1 | 1,153934 | 8,88404 | 0,046754 | *Acipenser ruthenus* genome assembly, chromosome: 32 | SI / histone H2A |
| GICD01089733.1 | 1,210297 | 7,82232 | 0,046754 | *Acipenser ruthenus* genome assembly, chromosome: 56 | SI / histone H2B 3-like |
| GICD01005390.1 | 1,699931 | 0,177221 | 0,046754 | 26S proteasome non-ATPase regulatory subunit 2 | NO ORF |
| GICD01074858.1 | 0,84345 | 7,793524 | 0,047584 | small ribosomal subunit protein eS26, mRNA | SI / glutathione reductase, mitochondrial |
| GICD01056685.1 | 1,021163 | 0,853664 | 0,047584 | BUD13 homolog, mRNA | SI / BUD13-like protein isoform X2 |
| GICD01048597.1 | 1,112064 | 3,51511 | 0,047584 | C-C motif chemokine 25-like, transcript variant X2 | SI / C-C motif chemokine 25-like |
| GICD01041939.1 | 1,016968 | 10,10985 | 0,04771 | *Acipenser ruthenus* genome assembly, chromosome: 45 | NO ORF |
