# Supplementary material for: Sex-specific gonadal transcriptome during early development of Siberian sturgeon
Source: Biol Sex Differ. 2026 Feb 2;17:17. doi: 10.1186/s13293-025-00810-8 (PMC12866037; doi:10.1186/s13293-025-00810-8)
Supplement: Supplementary file 6 — Supplementary Material 6 [file 13293_2025_810_MOESM6_ESM.docx]

Additional file 6. Male differentially expressed contigs

| NCBI reference | Log2FC | logCPM | FDR | Id Blastn | ORF / Id Blastp |
| --- | --- | --- | --- | --- | --- |
| GICD01008409.1_1-1800 | -1,529236 | 1,984467 | 1,38E-07 | T-box transcription factor TBX1-like (Tbx1) | SI / TBX1 factor |
| GICD01083699.1 | -2,017803134 | -0,532102263 | 3,59E-06 | Perilipin 1 like, transcript variant 2 | SI / perilipin-1 isoform X1 |
| GICD01068934.1 | -1,774617807 | 1,058260032 | 4,69E-05 | Neurexin 3, variant X39 | SI / neurexin-3 isoform X32 |
| GICD01019619.1 | -3,252234678 | -1,336299696 | 1,05E-03 | chitin synthase Chs-2 like | SI / chitin synthase Chs-2 like |
| GICD01001756.1 | -1,536115957 | -0,188108378 | 1,11E-03 | *Acipenser ruthenus* genome assembly, chromosome: 21 | NO ORF |
| GICD01036705.1 | -3,918490864 | -1,922582395 | 1,21E-03 | neprilysin-like, transcript variant X3 | NO ORF |
| GICD01043896.1 | -1,660580644 | 0,631914112 | 1,21E-03 | neurexin-3, transcript variant X41, mRNA | SI / neurexin-3 isoform X19 |
| GICD01053194.1 | -4,713563969 | 11,08234973 | 1,57E-03 | transmembrane protein 94 (tmem94), tr | NO ORF |
| GICD01000835.1 | -4,706586162 | 10,10352449 | 2,18E-03 | Acipenser ruthenus genome assembly, chromosome: 6 | NO ORF |
| GICD01008514.1 | -4,121827439 | 5,868776564 | 2,18E-03 | Acipenser ruthenus genome assembly, chromosome: 39 | NO ORF |
| GICD01008990.1 | -4,225845988 | 6,791503223 | 2,38E-03 | transmembrane protein 94 (tmem94), transcript variant X6, mRNA | NO ORF |
| GICD01086044.1 | -3,944996444 | 4,514961318 | 2,38E-03 | Acipenser ruthenus genome assembly, chromosome: 10 | NO ORF |
| GICD01031214.1 | -1,027327058 | 2,196726074 | 2,78E-03 | T-box transcription factor TBX1, transcript variant X3, mRNA | SI / T-box transcription factor TBX1-like isoform X3 |
| GICD01054378.1 | -1,420346868 | 2,710774727 | 2,93E-03 | No hay resultado | NO ORF |
| GICD01070285.1 | -3,11816535 | 1,997674019 | 3,62E-03 | Acipenser ruthenus genome assembly, chromosome: 6 | SI / hypothetical protein EGW08_016480 |
| GICD01023139.1 | -2,039308561 | -1,198839199 | 4,18E-03 | Acipenser ruthenus genome assembly, chromosome: 34 | SI / adhesion G protein-coupled receptor E3-like |
| GICD01015214.1 | -4,513834239 | 4,718967862 | 4,86E-03 | carboxypeptidase A1-like, mRNA | SI / carboxypeptidase A1-like |
| GICD01014422.1 | -3,454356449 | 2,04249569 | 4,86E-03 | UPF0606 protein KIAA1549-like, transcript variant X8, mRNA | NO ORF |
| GICD01046436.1 | -1,225681716 | 0,379457643 | 4,86E-03 | *Acipenser ruthenus* genome assembly, chromosome: 11 | NO ORF |
| GICD01009223.1 | -2,985308661 | -1,236099183 | 5,17E-03 | sodium/hydrogen exchanger 3-like, transcript variant X2, mRNA | SI / sodium/hydrogen exchanger 3-like isoform X2 |
| GICD01054640.1 | -3,234040385 | 6,506861468 | 5,17E-03 | hatching enzyme 1.2-like (LOC117432280), mRNA | SI / hatching enzyme 1.2-like |
| GICD01062130.1 | -8,391145134 | -0,867552004 | 5,82E-03 | fibrinogen gamma chain (fgg), mRNA | SI / Fibrinogen gamma chain |
| GICD01008640.1 | -5,102867866 | 4,778018337 | 5,82E-03 | bile salt-activated lipase-like, mRNA | SI / bile salt-activated lipase-like |
| GICD01034190.1 | -3,872296885 | 2,963232008 | 5,82E-03 | Acipenser ruthenus genome assembly, chromosome: 39 | SI / hypothetical protein AOXY_G3965 |
| GICD01068604.1 | -1,243241924 | 0,402898187 | 5,95E-03 | solute carrier family 10 member 1 (slc10a1), transcript variant X2, mRNA | SI / hepatic sodium/bile acid cotransporter |
| GICD01059159.1 | -2,746514104 | -0,811364675 | 6,35E-03 | putative defense protein 3, mRNA | SI / putative defense protein 3 |
| GICD01086977.1 | -6,548083133 | 6,919828767 | 6,41E-03 | trypsin-3-like, mRNA | SI / trypsin-3-like |
| GICD01071611.1 | -1,276433003 | 1,361094015 | 7,71E-03 | kyphoscoliosis peptidase, mRNA | SI / Kyphoscoliosis peptidase |
| GICD01066825.1 | -1,872739642 | -0,100283894 | 9,14E-03 | Na(+)/H(+) exchange regulatory cofactor NHE-RF3-like | NO ORF |
| GICD01080672.1 | -5,352563837 | -3,225294037 | 1,07E-02 | alpha-N-acetylgalactosaminidase-like | NO ORF |
| GICD01080771.1 | -3,659154426 | 3,615664799 | 1,07E-02 | genome assembly, chromosome: 12 | NO ORF |
| GICD01052182.1 | -1,424215632 | 3,189485323 | 1,17E-02 | genome assembly, chromosome: 43 | NO ORF |
| GICD01028154.1 | -2,747760585 | 7,216811936 | 1,42E-02 | genome assembly, chromosome: 30 | NO ORF |
| GICD01017126.1 | -3,617736976 | -2,56149142 | 1,45E-02 | homeobox protein vent1-like, transcript variant X3, mRNA | NO ORF |
| GICD01022527.1 | -4,916306545 | 2,907626808 | 1,49E-02 | carboxypeptidase A1-like, transcript variant X2, mRNA | SI / carboxypeptidase A1-like isoform X1 |
| GICD01065547.1 | -2,40263591 | 4,323533584 | 1,49E-02 | matrix metalloproteinase-9-like, mRNA | SI / matrix metalloproteinase-9-like |
| GICD01006691.1 | -3,303823193 | -1,860741221 | 1,68E-02 | extracellular calcium-sensing receptor-like, mRNA | SI / extracellular calcium-sensing receptor-like |
| GICD01000206.1 | -4,676452703 | 3,001227325 | 1,69E-02 | serine protease 1-like, mRNA | SI / serine protease 1-like |
| GICD01030534.1 | -2,125393974 | 1,000180299 | 1,69E-02 | Acipenser ruthenus genome assembly, chromosome: 42 | SI / hypothetical protein HHUSO_G4208 |
| GICD01077289.1 | -1,518651753 | 1,546065189 | 1,69E-02 | ammonium transporter Rh type A-like, mRNA | SI / ammonium transporter Rh type A-like [Huso huso] |
| GICD01069196.1 | -3,772293876 | 3,91363825 | 1,94E-02 | inactive pancreatic lipase-related protein 1-like, mRNA | SI / inactive pancreatic lipase-related protein 1-like |
| GICD01049333.1 | -1,458291144 | 1,210203609 | 2,19E-02 | microtubule-associated protein RP/EB family member 2-like (LOC117400421), transcript variant X3 | NO ORF |
| GICD01030365.1 | -0,807532425 | 2,895564779 | 2,55E-02 | Acipenser ruthenus genome assembly, chromosome: 9 | NO ORF |
| GICD01017837.1 | -2,182152744 | 1,735086196 | 2,58E-02 | pleckstrin homology domain-containing family H member 2-like | NO ORF |
| GICD01048432.1 | -0,974312654 | 1,693840411 | 3,32E-02 | Acipenser ruthenus genome assembly, chromosome: 40 | NO ORF |
| GICD01089001.1 | -1,011342999 | 2,390388822 | 3,49E-02 | actin filament-associated protein 1-like, transcript variant X4, mRNA | NO ORF |
| GICD01089820.1 | -0,967512931 | 1,839551342 | 3,82E-02 | Acipenser ruthenus genome assembly, chromosome: 43 | NO ORF |
| GICD01009610.1 | -1,171884647 | 0,387160469 | 3,82E-02 | Acipenser ruthenus genome assembly, chromosome: 21 | NO ORF |
| GICD01086954.1 | -1,183197352 | 1,341833046 | 4,05E-02 | Acipenser ruthenus genome assembly, chromosome: 43 | NO ORF |
| GICD01057816.1 | -1,686174642 | 3,610697497 | 4,51E-02 | cytochrome P450 1A1, mRNA | SI / cytochrome P450 1A1 |
| GICD01034736.1 | -4,229544577 | 1,736394127 | 4,68E-02 | bile salt-activated lipase-like (LOC117396792), mRNA | SI / bile-salt activated lipase |
| GICD01013799.1 | -1,984375014 | -1,707598355 | 4,68E-02 | phospholipidtransfer protein-like, transcript variant X2, mRNA | NO ORF |
| GICD01027021.1 | -1,02116599 | 1,656251417 | 4,68E-02 | Acipenser ruthenus genome assembly, chromosome: 6 | NO ORF |
| GICD01039412.1 | -1,014399349 | 1,306773174 | 4,68E-02 | Acipenser ruthenus genome assembly, chromosome: 36 | NO ORF |
| GICD01083671.1 | -1,700257274 | 1,414664976 | 4,73E-02 | interleukin-6 receptor subunit beta-like, transcript variant X3, mRNA | SI / interleukin-6 receptor subunit beta-like |
| GICD01021264.1 | -1,174071582 | 0,448959646 | 4,77E-02 | Acipenser ruthenus genome assembly, chromosome: 40 | SI / zinc finger protein 252-like isoform X2 |
| GICD01084215.1 | -1,43026781 | 0,440481155 | 4,92E-02 | sodium channel subunit beta-4-like, transcript variant X4, mRNA | NO ORF |
