# Supplementary material for: Sex-specific gonadal transcriptome during early development of Siberian sturgeon
Source: Biol Sex Differ. 2026 Feb 2;17:17. doi: 10.1186/s13293-025-00810-8 (PMC12866037; doi:10.1186/s13293-025-00810-8)
Supplement: Supplementary file 7 — Supplementary Material 7 [file 13293_2025_810_MOESM7_ESM.docx]

Additional file 7. Characteristics of coding contigs differentially expressed in females

| NCBI contig reference n° | Gen “Symbol” | Log_2_FC | FDR | Id Blastn | ORF / Id Blastp |
| --- | --- | --- | --- | --- | --- |
| GICD01088227.1 | *hsd17b1* | 3,110912 | 2,18E-11 | hsd17b1 | hsd17b1 |
| GICD01082292.1 | *chrna7* | 2,181414 | 9,16E-09 | neuronal acetylcholine receptor subunit alpha-7 like | neuronal acetylcholine receptor subunit alpha-7 like |
| GICD01027714.1 | *ier2* | 1,514963 | 4,24E-07 | immediate early response gene 2 protein-like | immediate early response gene 2 protein-like |
| GICD01064951.1 | *diras2* | 2,405916 | 8,92E-07 | GTP-binding protein Di-Ras2 | GTP-binding protein Di-Ras2 |
| GICD01077530.1 | *c-fos* | 3,013134 | 8,92E-07 | Protein c-Fos like | proto-oncogene c-Fos-like |
| GICD01090265.1 | *cyp19a1a* | 1,958954 | 9,17E-07 | aromatase (*cyp19a1*) | Aromatase (*cyp19a1*) |
| GICD01087373.1 | *trhr* | 1,765854 | 2,1E-06 | thyrotropin-releasing hormone receptor-like | Thyrotropin-releasing hormone receptor |
| GICD01023208.1 | *foxl2* | 2,635137 | 1,57E-05 | forkhead box protein L2, (*foxl2*) | forkhead box protein L2 (*foxl2*) |
| GICD01059572.1 | *jun-b* | 1,821218 | 0,000213 | transcription factor JunB-like | transcription factor JunB isoform X1 |
| GICD01087320.1 | *tmem271* | 1,38849 | 0,006129 | transmembrane protein 271-like mRNA | transmembrane protein 271-like mRNA |
| GICD01032392.1 | *tonsl* | 1,37497 | 0,006423 | tonsoku-like protein, mRNA | Tonsoku-like protein |
| GICD01084445.1 | *egr1* | 1,144446 | 0,007341 | early growth response protein 1-like | early growth response protein 1-like |
| GICD01059511.1 | *ier2* | 1,449407 | 0,007341 | immediate early response gene 2 protein-like | immediate early response gene 2 protein-like |
| GICD01007426.1 | *mhc class I antigen* | 4,642036 | 0,009067 | MHC class 1 alpha antigen | MHC class I alpha antigen |
| GICD01089620.1 | *aanat* | 3,078826 | 0,009078 | serotonin N-acetyltransferase-like | serotonin N-acetyltransferase-like |
| GICD01078088.1 | *c-fos* | 2,15909 | 0,014769 | protein c-Fos-like, mRNA | proto-oncogene c-Fos-like isoform X1 |
| GICD01009598.1 | *?* | 0,955002 | 0,01866 | Uncharacterized protein, mRNA | uncharacterized protein LOC131735831 |
| GICD01009655.1 | *h2 k1* | 8,197664 | 0,021374 | MHC class lachain (Acsi-UBA) | H-2 class I histocompatibility antigen |
| GICD01001726.1 | *h4c7* | 1,067252 | 0,021872 | *Acipenser ruthenus* genome assembly, chromosome: 32 | histone H4-like |
| GICD01000328.1 | *?* | 1,174873 | 0,025759 | histone H2A, mRNA | uncharacterized protein LOC121514829 |
| GICD01066315.1 | *h4c7* | 0,929594 | 0,030163 | *Acipenser ruthenus* genome assembly, chromosome: 55 | histone H4-like |
| GICD01015861.1 | *asrgl1* | 1,330658 | 0,032135 | L-asparaginase-like, mRNA | L-asparaginase-like |
| GICD01010688.1 | *?* | 1,528112 | 0,033206 | *Acipenser ruthenus* genome assembly, chromosome: 51 | hypothetical protein EOD39_19946 |
| GICD01073114.1 | *h2ac11* | 1,35344 | 0,038297 | histone H2A, mRNA | H2A protein |
| GICD01030746.1 | *h2ac* | 0,808082 | 0,04469 | *Acipenser ruthenus* genome assembly, chromosome: 20 | histone H2A-like |
| GICD01066742.1 | *mpst* | 0,851523 | 0,046754 | mercaptopyruvate sulfurtransferase (mpst), mRNA | 3-mercaptopyruvate sulfurtransferase |
| GICD01059541.1 | *rnf227* | 0,92957 | 0,046754 | RING finger protein 227-like, mRNA | RING finger protein 227 |
| GICD01080907.1 | *h2ac* | 1,153934 | 0,046754 | *Acipenser ruthenus* genome assembly, chromosome: 32 | histone H2A |
| GICD01089733.1 | *h2b-3* | 1,210297 | 0,046754 | *Acipenser ruthenus* genome assembly, chromosome: 56 | histone H2B 3-like |
| GICD01074858.1 | *gsr* | 0,84345 | 0,047584 | small ribosomal subunit protein eS26, mRNA | glutathione reductase, mitochondrial |
| GICD01056685.1 | *bud13* | 1,021163 | 0,047584 | BUD13 homolog, mRNA | BUD13-like protein isoform X2 |
| GICD01048597.1 | *ccl25* | 1,112064 | 0,047584 | C-C motif chemokine 25-like, transcript variant X2 | C-C motif chemokine 25-like |
