# Supplementary material for: Sex-specific gonadal transcriptome during early development of Siberian sturgeon
Source: Biol Sex Differ. 2026 Feb 2;17:17. doi: 10.1186/s13293-025-00810-8 (PMC12866037; doi:10.1186/s13293-025-00810-8)
Supplement: Supplementary file 8 — Supplementary Material 8 [file 13293_2025_810_MOESM8_ESM.docx]

Additional file 8. Characteristics of coding contigs differentially expressed in males

| NCBI contig reference n° | Gen “Symbol” | Log_2_FC | FDR | Id Blastn | ORF / Id Blastp |
| --- | --- | --- | --- | --- | --- |
| GICD01008409.1 | *tbx1* | -1,529236 | 1,38E-07 | T-boxtranscription factor TBX1-like (Tbx1) | TBX1 factor |
| GICD01083699.1 | *plin1* | -2,017803 | 3,59E-06 | Perilipin 1 like, transcript variant 2 | perilipin-1 isoform X1 |
| GICD01068934.1 | *nrxn3* | -1,77461 | 4,69E-05 | Neurexin 3, variant X39 | neurexin-3 isoform X32 |
| GICD01019619.1 | *chs2* | -3,25223 | 1,05E-03 | chitin synthase Chs-2 like | chitin synthase Chs-2 like |
| GICD01043896.1 | *nrxn3* | -1,66058 | 1,21E-03 | neurexin-3, transcript variant X41, mRNA | neurexin-3 isoform X19 |
| GICD01031214.1 | *tbx1* | -1,02732 | 2,78E-03 | T-box transcription factor TBX1, transcript variant X3, mRNA | T-box transcription factor TBX1-like isoform X3 |
| GICD01070285.1 | *?* | -3,11816 | 3,62E-03 | Acipenser ruthenus genome assembly, chromosome: 6 | hypothetical protein EGW08_016480 |
| GICD01023139.1 | *adgre3* | -2,03930 | 4,18E-03 | Acipenser ruthenus genome assembly, chromosome: 34 | adhesion G protein-coupled receptor E3-like |
| GICD01015214.1 | *cpa1* | -4,51383 | 4,86E-03 | carboxypeptidase A1-like, mRNA | carboxypeptidase A1-like |
| GICD01009223.1 | *slc9a3* | -2,98530 | 5,17E-03 | sodium/hydrogen exchanger 3-like, transcript variant X2, mRNA | sodium/hydrogen exchanger 3-like isoform X2 |
| GICD01054640.1 | *he1.2* | -3,23404 | 5,17E-03 | hatching enzyme 1.2-like (LOC117432280), mRNA | hatching enzyme 1.2-like |
| GICD01062130.1 | *fgg* | -8,39114 | 5,82E-03 | fibrinogen gamma chain (fgg), mRNA | Fibrinogen gamma chain |
| GICD01008640.1 | *cel* | -5,10286 | 5,82E-03 | bile salt-activated lipase-like, mRNA | bile salt-activated lipase-like |
| GICD01034190.1 | *?* | -3,87229 | 5,82E-03 | Acipenser ruthenus genome assembly, chromosome: 39 | hypothetical protein AOXY_G3965 |
| GICD01068604.1 | *slc10a1* | -1,24324 | 5,95E-03 | solute carrier family 10 member 1 (slc10a1), transcript variant X2, mRNA | hepatic sodium/bile acid cotransporter |
| GICD01059159.1 | *dfp3* | -2,746514 | 6,35E-03 | putative defense protein 3, mRNA | putative defense protein 3 |
| GICD01086977.1 | *prss3* | -6,548083 | 6,41E-03 | trypsin-3-like, mRNA | trypsin-3-like |
| GICD01071611.1 | *ky* | -1,276433 | 7,71E-03 | kyphoscoliosis peptidase, mRNA | Kyphoscoliosis peptidase |
| GICD01022527.1 | *cpa1* | -4,91630 | 1,49E-02 | carboxypeptidase A1-like, transcript variant X2, mRNA | carboxypeptidase A1-like isoform X1 |
| GICD01065547.1 | *mmp9* | -2,40263 | 1,49E-02 | matrix metalloproteinase-9-like, mRNA | matrix metalloproteinase-9-like |
| GICD01006691.1 | *casr* | -3,30382 | 1,68E-02 | extracellular calcium-sensing receptor-like, mRNA | extracellular calcium-sensing receptor-like |
| GICD01000206.1 | *prss1* | -4,67645 | 1,69E-02 | serine protease 1-like, mRNA | serine protease 1-like |
| GICD01030534.1 | *?* | -2,12539 | 1,69E-02 | Acipenser ruthenus genome assembly, chromosome: 42 | hypothetical protein HHUSO_G4208 |
| GICD01077289.1 | *rhag* | -1,51865 | 1,69E-02 | ammonium transporter Rh type A-like, mRNA | ammonium transporter Rh type A-like [Huso huso] |
| GICD01069196.1 | *Pnliprp1* | -3,77229 | 1,94E-02 | inactive pancreatic lipase-related protein 1-like, mRNA | inactive pancreatic lipase-related protein 1-like |
| GICD01057816.1 | *cyp1a1* | -1,68617 | 4,51E-02 | cytochrome P450 1A1, mRNA | cytochrome P450 1A1 |
| GICD01034736.1 | *cel* | -4,22954 | 4,68E-02 | bile salt-activated lipase-like (LOC117396792), mRNA | bile-salt activated lipase |
| GICD01083671.1 | *il6st* | -1,70025 | 4,73E-02 | interleukin-6 receptor subunit beta-like, transcript variant X3, mRNA | interleukin-6 receptor subunit beta-like |
| GICD01021264.1 | *zfn252p* | -1,17407 | 4,77E-02 | Acipenser ruthenus genome assembly, chromosome: 40 | zinc finger protein 252-like isoform X2 |
