# Supplementary material for: Sex-specific gonadal transcriptome during early development of Siberian sturgeon
Source: Biol Sex Differ. 2026 Feb 2;17:17. doi: 10.1186/s13293-025-00810-8 (PMC12866037; doi:10.1186/s13293-025-00810-8)
Supplement: Supplementary file 9 — Supplementary Material 9 [file 13293_2025_810_MOESM9_ESM.docx]

Additional file 9. Selected female significant GO terms linked to sex determination and differentiation processes

| GO.ID | Term | Process | ajusted p-value |
| --- | --- | --- | --- |
| GO:0051412 | response to corticosterone | Biological Process | 2.90E-05 |
| GO:0051385 | response to mineralocorticoid | Biological Process | 1.05E-04 |
| GO:0030186 | melatonin metabolic process | Biological Process | 1.43E-03 |
| GO:0061370 | testosterone biosynthetic process | Biological Process | 1.43E-03 |
| GO:0042446 | hormone biosynthetic process | Biological Process | 1.69E-03 |
| GO:0006703 | estrogen biosynthetic process | Biological Process | 2.58E-03 |
| GO:0120178 | steroid hormone biosynthetic process | Biological Process | 2.58E-03 |
| GO:0060136 | embryonic process involved in female pregnancy | Biological Process | 2.58E-03 |
| GO:0032570 | response to progesterone | Biological Process | 6.32E-03 |
| GO:0042445 | hormone metabolic process | Biological Process | 8.50E-03 |
| GO:0071372 | cellular response to follicle-stimulating hormone | Biological Process | 8.75E-03 |
| GO:0008210 | estrogen metabolic process | Biological Process | 8.75E-03 |
| GO:0031960 | response to corticosteroid | Biological Process | 8.75E-03 |
| GO:0030237 | female sex determination | Biological Process | 8.75E-03 |
| GO:0032354 | response to follicle-stimulating hormone | Biological Process | 1.10E-02 |
| GO:0006710 | androgen catabolic process | Biological Process | 1.26E-02 |
| GO:0048545 | response to steroid hormone | Biological Process | 1.42E-02 |
| GO:0008207 | C21-steroid hormone metabolic process | Biological Process | 1.59E-02 |
| GO:0060014 | granulosa cell differentiation | Biological Process | 1.85E-02 |
| GO:0006694 | steroid biosynthetic process | Biological Process | 2.49E-02 |
| GO:0034698 | response to gonadotropin | Biological Process | 2.75E-02 |
| GO:0060986 | endocrine hormone secretion | Biological Process | 2.75E-02 |
| GO:0033686 | positive regulation of luteinizing hormone | Biological Process | 2.75E-02 |
| GO:0030187 | melatonin biosynthetic process | Biological Process | 2.99E-02 |
| GO:0033684 | regulation of luteinizing hormone secretion | Biological Process | 2.99E-02 |
| GO:2000182 | regulation of progesterone biosynthetic process | Biological Process | 2.99E-02 |
| GO:0032275 | luteinizing hormone secretion | Biological Process | 3.60E-02 |
| GO:0007530 | sex determination | Biological Process | 3.74E-02 |
| GO:0042698 | ovulation cycle | Biological Process | 3.90E-02 |
| GO:0006701 | progesterone biosynthetic process | Biological Process | 3.90E-02 |
| GO:0032276 | regulation of gonadotropin secretion | Biological Process | 3.90E-02 |
| GO:0035938 | estradiol secretion | Biological Process | 3.90E-02 |
| GO:0019101 | female somatic sex determination | Biological Process | 4.08E-02 |
| GO:0008202 | steroid metabolic process | Biological Process | 4.08E-02 |
| GO:0032274 | gonadotropin secretion | Biological Process | 4.47E-02 |
| GO:0035976 | transcription factor AP-1 complex | Cellular Component | 1.25E-05 |
| GO:0001216 | DNA-binding transcription activator activity | Molecular Function | 1.17E-02 |
| GO:0001228 | DNA-binding transcription activator activity RNA polymerase II-specific | Molecular Function | 1.17E-02 |
