# Supplementary material for: Sex-specific gonadal transcriptome during early development of Siberian sturgeon
Source: Biol Sex Differ. 2026 Feb 2;17:17. doi: 10.1186/s13293-025-00810-8 (PMC12866037; doi:10.1186/s13293-025-00810-8)
Supplement: Supplementary file 10 — Supplementary Material 10 [file 13293_2025_810_MOESM10_ESM.docx]

Additional file 10*.* Male significant GO terms linked to sex determination and differentiation processes

| GO.ID | Term | Process | p_adj |
| --- | --- | --- | --- |
| GO:0006706 | steroid catabolic process | Biological Process | 2,69E-04 |
| GO:0001523 | retinoid metabolic process | Biological Process | 1,37E-03 |
| GO:0034633 | retinol transport | Biological Process | 1,37E-03 |
| GO:0030301 | cholesterol transport | Biological Process | 2,59E-03 |
| GO:0042572 | retinol metabolic process | Biological Process | 2,59E-03 |
| GO:0006707 | cholesterol catabolic process | Biological Process | 2,59E-03 |
| GO:0033189 | response to vitamin A | Biological Process | 7,39E-03 |
| GO:0042445 | hormone metabolic process | Biological Process | 1,51E-02 |
| GO:0010817 | regulation of hormone levels | Biological Process | 2,06E-02 |
| GO:0008202 | steroid metabolic process | Biological Process | 2,20E-02 |
| GO:0002138 | retinoic acid biosynthetic process | Biological Process | 3,07E-02 |
| GO:0009062 | fatty acid catabolic process | Biological Process | 3,15E-02 |
| GO:0008203 | cholesterol metabolic process | Biological Process | 3,16E-02 |
| GO:0042573 | retinoic acid metabolic process | Biological Process | 4,29E-02 |
| GO:0004806 | triglyceride lipase activity | Molecular Function | 4,17E-04 |
| GO:0050253 | retinyl-palmitate esterase activity | Molecular Function | 4,17E-04 |
| GO:0008237 | metallopeptidase activity | Molecular Function | 3,70E-03 |
